# Inflammasome activation in peritumoral astrocytes is a key player in breast cancer brain metastasis development

**DOI:** 10.1186/s40478-023-01646-2

**Published:** 2023-09-25

**Authors:** Ádám Mészáros, Kinga Molnár, Csilla Fazakas, Bernát Nógrádi, Adél Lüvi, Tamás Dudás, László Tiszlavicz, Attila Elek Farkas, István Adorján Krizbai, Imola Wilhelm

**Affiliations:** 1 Institute of Biophysics, Biological Research Centre, ELKH (Eötvös Loránd Research Network), Temesvári Krt. 62, 6726 Szeged, Hungary; 2 Doctoral School of Biology, University of Szeged, Szeged, Hungary; 3 Theoretical Medicine Doctoral School, University of Szeged, Szeged, Hungary; 4 Department of Pathology, University of Szeged, Szeged, Hungary; 5 Institute of Life Sciences, Vasile Goldiş Western University of Arad, Arad, Romania; 6 Present Address: Department of Neurology, University of Szeged, Szeged, Hungary

**Keywords:** Brain metastasis, IL-1β, Inflammatory microenvironment, NLRP3 inflammasome, Peritumoral astrocytes, Triple-negative breast cancer

## Abstract

Inflammasomes, primarily responsible for the activation of IL-1β, have emerged as critical regulators of the tumor microenvironment. By using in vivo and in vitro brain metastasis models, as well as human samples to study the role of the NLRP3 inflammasome in triple-negative breast cancer (TNBC) brain metastases, we found NLRP3 inflammasome components and IL-1β to be highly and specifically expressed in peritumoral astrocytes. Soluble factors from TNBC cells induced upregulation and activation of NLRP3 and IL-1β in astrocytes, while astrocyte-derived mediators augmented the proliferation of metastatic cells. In addition, inhibition of NLRP3 inflammasome activity using MCC950 or dampening the downstream effect of IL-1β prevented the proliferation increase in cancer cells. In vivo, MCC950 reduced IL-1β expression in peritumoral astrocytes, as well as the levels of inflammasome components and active IL-1β. Most importantly, significantly retarded growth of brain metastatic tumors was observed in mice treated with MCC950. Overall, astrocytes contribute to TNBC progression in the brain through activation of the NLRP3 inflammasome and consequent IL-1β release. We conclude that pharmacological targeting of inflammasomes may become a novel strategy in controlling brain metastatic diseases.

**Supplementary Information:**

The online version contains supplementary material available at 10.1186/s40478-023-01646-2.

## Introduction

Triple-negative breast cancer (TNBC) is one of the most common causes of brain metastases in women [27]. Due to the lack of effective treatment options, these tumors have one of the most dismal prognoses [31]. Therefore, the identification of therapeutic targets—either on tumor cells or on elements of the microenvironment—is urgently needed. As a key regulator of tumor progression, the microenvironment represents a promising site in this respect [45].

The brain metastatic environment consists of vascular cells (endothelial cells and pericytes), glial cells, neurons, and tumor-infiltrating immune cells [29, 48]. In addition, the vascular basement membrane—secreted in a fairly large part by pericytes [36, 50]—provides essential survival clues for metastatic cells [5, 52].

Among the cellular elements of the tumor environment in the brain, endothelial cells provide chemoprotection to breast cancer cells [19], pericytes increase their proliferation [36], and astrocytes have a Janus-faced attitude toward them [56]. By secreting plasminogen activators, astroglia activate plasmin, which cleaves both Fas-ligand to destroy cancer cells and L1 cell adhesion molecule (L1-CAM) to block adhesion of the tumor cells to the vessel walls [52]. On the other hand, astrocytes establish direct contacts with metastatic cells to upregulate survival genes in them and to provide them with chemoresistant features [6, 23]. In addition, astrocytes release protumoral factors, including exosomes, proteases and proinflammatory cytokines [6, 53, 62].

The inflammatory environment—provided by astrocytes, microglia and infiltrating immune cells—has been shown to facilitate metastatic growth [10]. Among proinflammatory cytokines, interleukin-1β (IL-1β) is one of the most potent and has opposing effects on the development of tumors and metastases, either promoting or inhibiting them [2, 51]. IL-1β is mainly secreted in an inflammasome-dependent manner. Among different types of inflammasomes, the nucleotide-binding and oligomerization domain, leucine rich repeat and pyrin domain-containing protein 3 (NLRP3) inflammasome is one of the best characterized, gaining functionality in two steps, priming and activation [49]. Priming (i.e., increased transcription and translation, as well as posttranscriptional modifications) of inflammasome components may be induced by either microorganisms or damage-associated molecular patterns, such as cytokines [33]. The activation itself means assembly of the functional inflammasome comprised of the NLRP3 protein, the apoptosis-associated speck-like protein containing a caspase activation and recruitment domain (ASC) adaptor protein and pro-caspase-1. Oligomerization of ASC into a macromolecular aggregate called the ASC speck recruits pro-caspase-1 and activates it, which in turn cleaves pro-IL-1β into active IL-1β [3].

In the central nervous system (CNS), the NLRP3 inflammasome is activated in aging [58] and in several pathological conditions [34], including stroke [61], traumatic brain injury [40] and neurodegenerative disorders, such as Alzheimer’s disease [20], multiple sclerosis [16] and Parkinson’s disease [41] but also in injuries of peripheral nerves [37]. The NLRP3 inflammasome may become activated in different cells of the CNS, including vascular cells [24], neurons [37], oligodendrocytes [63] or ependymal cells [64], but the main cell types involved are microglia and astrocytes [26].

In the present study, we aimed to understand whether activation of the NLRP3 inflammasome is involved in the pathogenesis of brain metastasis formation. In addition, we wanted to determine the cell type responsible for possible IL-1β release and its effects on metastatic breast cancer cells at the early stage of brain colonization.

## Materials and methods

### Cell culture

#### Culture of human astrocytes

Human astrocytes (HAs, ScienCell Research Laboratories, Carlsbad, CA, USA) were cultured on poly-l-lysine-coated dishes in astrocyte medium supplemented with 5% fetal bovine serum (FBS), astrocyte growth supplements and penicillin/streptomycin (all from ScienCell Research Laboratories) and were used between passages 2 and 5.

#### Isolation and culture of mouse astrocytes

Primary mouse astrocytes (MAs) were isolated from the brains of 1- to 2-day-old BALB/c mouse pups. After removal of the meninges, brains were mechanically triturated in low-glucose Dulbecco’s modified Eagle’s medium (DMEM; Thermo Fisher Scientific, Waltham, MA, USA), and the suspension was filtered through a 40 µm pore size cell strainer (Thermo Fisher Scientific). Astrocytes were seeded onto poly-l-lysine-coated dishes and were grown in DMEM supplemented with 10% FBS (Thermo Fisher Scientific). After reaching confluence, cells were used as primary cells (passage number 0) or at passage number 1.

#### Culture of tumor cells

The human TNBC cells—the parental cell line MDA-MB-231-TGL (MDA-231-HSV1-TK/GFP/Fluc, MDA-TGL for short; RRID:CVCL_VR35) and the brain-seeking MDA-MB-231-BrM2 (abbreviated as MDA-BrM2; RRID:CVCL_VR36) cells—were obtained from Dr. Joan Massagué at Memorial Sloan Kettering Cancer Center (New York, NY, USA). The cells were kept in DMEM supplemented with 10% FBS (Thermo Fisher Scientific). The cells were authenticated by analysis of highly variable short tandem repeat (STR) markers using Applied Biosystems™ GeneMapper™ Software 6 (Thermo Fisher Scientific).

The mouse tdTomato-expressing TNBC cells (4T1-tdTomato, 4T1-tdT)—described elsewhere [17], originating from 4T1 cells (RRID:CVCL_0125) after transfection with the pcDNA3.1(+)/Luc2 = tdT plasmid—were cultured in Roswell Park Memorial Institute (RPMI) 1640 medium supplemented with 5% FBS and G418 (all from Thermo Fisher Scientific). 4T1-tdT cells were authenticated by Microsynth (Balgach, Switzerland) using analysis of highly polymorphic STR loci.

All cell types were regularly tested for mycoplasma infection with a MycoAlert Mycoplasma Detection Kit (Lonza, Basel, Switzerland) and were maintained at 37 °C in a humid incubator with 5% CO_2_ in air.

#### Preparation of conditioned media

To obtain HA-conditioned medium (HA-CM), we cultured HAs in poly-l-lysine-coated 6-well plates until 90% confluence. Cells received fresh HA medium for 2 days, and then HA-CM was collected.

MAs were cultured in the same way as HAs until confluence. MA culture medium was changed to a 1:1 mixture of serum-free RPMI and MA complete medium. MA-conditioned medium (MA-CM) was collected after 48 h.

MDA-TGL or MDA-BrM2 cells (5 × 10^5^) were plated into 6 cm dishes in their own medium. The next day, the culture medium was changed to HA complete medium. MDA-TGL CM or MDA-BrM2 CM were collected after 48 h.

4T1-tdT cells were plated as described above. After 24 h, the culture medium was replaced with a 1:1 mixture of serum-free RPMI and MA complete medium. 4T1-tdT CM was collected after 48 h.

HAs were plated into poly-l-lysine-coated 6-well plates in their own culture medium. At 90% confluence, the culture medium was replaced with a 1:1 mixture of fresh HA complete medium and MDA-TGL CM or MDA-BrM2 CM. Activated HA-conditioned media (act. HA-CM) were collected after 48 h.

MAs were cultured in poly-l-lysine-coated 6-well plates. After reaching confluence, cells received a mixture of 1/4 serum free-RPMI, 1/4 MA complete medium and 1/2 4T1-tdT CM. Activated MA-conditioned medium (act. MA-CM) was collected after 48 h.

Nonconditioned medium was used as a negative control. After collection, all conditioned media were filtered through 0.2 µm pore size syringe filters to remove cellular debris.

Where indicated, conditioned media were collected from HAs or MAs treated with 1 µM MCC950 (InvivoGen, San Diego, CA, USA) for 2 days. Dimethyl sulfoxide (DMSO; Merck-Sigma, St. Louis, MO, USA) was used as a vehicle control.

#### Proliferation assays

To test the effect of IL-1β on the proliferation of breast cancer cells, 10^4^ MDA-TGL, MDA-BrM2 or 4T1-tdT cells were plated into 6-well plates in the presence or absence of 10 ng/ml recombinant IL-1β (ImmunoTools GmbH, Friesoythe, Germany). Medium and IL-1β treatment were refreshed after 2 days.

To understand the impact of astrocyte-secreted factors on the proliferation of breast cancer cells, 10^4^ MDA-TGL or MDA-BrM2 cells were cultured in 6-well plates in HA-CM or act. HA-CM for 4 days. Nonconditioned HA medium was used as a control. The medium was changed to freshly collected CM after 48 h. The same setup was established by using 4T1-tdT cells and MA-CM or act. MA-CM. The proliferative behavior of MDA-BrM2 cells and 4T1-tdT cells was also assessed in CM collected from HAs or MAs, respectively, which were incubated for 48 h with MCC950 to inhibit the NLRP3 inflammasome.

Moreover, after collection of HA-/MA-CM or act. HA-/MA-CM, where indicated, 2 µg of human or mouse IL-1β neutralizing antibodies were added to 1500 µl of medium to block cognate ligand‒receptor interactions. As a negative control, normal goat IgG was added to the medium at the same concentration as the neutralizing antibodies. Media were incubated with the antibodies for 30 min at room temperature before being used for the experiment.

Phase contrast images of the tumor cells were acquired with a Nikon Eclipse TE2000-U inverted microscope (Nikon, Tokyo, Japan) connected to a digital camera (ORCA-Fusion CMOS camera, Hamamatsu Photonics, Hamamatsu, Japan). Quantification of cells was performed manually using the Cell Counter plugin of FIJI (Fiji is just ImageJ, Max Planck Institute of Molecular Cell Biology and Genetics, Dresden, Germany) [43].

### Experimental animal surgery and treatments

Experiments were carried out on young adult (8–12 weeks old, 20 ± 3 g) female BALB/c mice (Charles River Laboratories, Wilmington, MA, USA) or FVB/Ant:TgCAG‐yfp_sb #27 mice (Venus mice, for short). Venus mice were kindly gifted by the Institute of Experimental Medicine, Budapest, Hungary. All mice were housed under standard conditions (12-h light/dark cycle, 23 ± 2 °C) with ad libitum access to regular chow and water.

For ex vivo experiments, brain metastases were generated as reported previously [36]. Briefly, once anesthetized with isoflurane (4% induction, 2% maintenance), BALB/c mice were inoculated with 10^6^ 4T1-tdT cells in 100 μl sterile Krebs–Ringer solution into the right common carotid artery, with the right external carotid artery transiently ligated. Upon completion of the injection, mice were divided into survival groups at 2, 5 and 7 days. These time points were chosen based on the onset before, during and after extravasation of breast cancer cells into the brain [17].

For determination of tumor size and microgliosis, Venus mice received 2 × 10^6^ 4T1‐tdT cells/200 µl intracardially, according to a method previously described [4]. Mice were sacrificed after 7 days.

Where indicated, animals in the 7-day postinjection group were administered an intraperitoneal injection of 10 mg/kg body weight MCC950 (InvivoGen) every day from day 4 to day 6 after cancer cell injection, i.e., starting from the day when diapedesis of brain metastatic breast cancer cells usually occurs in mouse models [17, 22, 30]. As a vehicle control for MCC950, another group of mice received DMSO in phosphate buffered saline (PBS) intraperitoneally. On the indicated days, mice were euthanized, and brains were harvested for further processing.

After surgery, animals were kept individually under the conditions described above. The survival status and body weight of tumor-bearing animals were monitored daily. The survival rate was 97–98%, and a maximum 5% weight loss was observed by the end of the postoperative period. The animals did not develop infection, wound dehiscence or internal bleeding and did not show chronic signs of pain. All experimental animals were randomly allocated by a blinded investigator to the particular treatment groups. During the experiments, no confounders were identified.

### Tissue fixation and sectioning

Human TNBC brain metastasis and control brain samples were obtained from the Department of Pathology, University of Szeged, Szeged, Hungary. The two brain metastasis samples were received from the neurosurgical resection of an occipital tumor of a 41 year-old, and a frontal tumor of a 39 year-old female patient. Primary tumors or extracranial metastases were not evaluated. Paraffin-embedded blocks of tissue specimens were cut into 5 μm thick sections for immunofluorescence staining.

4T1-tdT-bearing mice were transcardially perfused with PBS (0.1 M, pH = 7.4) and subsequently fixed with 4% paraformaldehyde (PFA) in PBS. Following postfixation and cryoprotection, 20 µm thick coronal brain sections were cut using a freezing microtome (Reichert‐Jung, Leica Biosystems, Wetzlar, Germany). Free-floating sections were stored in 10 mM PBS with 0.02% sodium-azide (Merck-Sigma) until further processing.

### Immunofluorescence staining, microscopy and quantification of the signals

#### Immunofluorescence staining

HAs grown in 12-well glass-bottomed microscope chambers were fixed with 4% PFA in PBS for 10 min. After three washing steps in PBS, the cells were blocked with 3% bovine serum albumin (BSA; VWR International, Radnor, PA, USA) in PBS containing 0.2% Triton X-100 (Merck-Sigma) for 1 h. Primary antibodies (Additional file 1: Table S1) were diluted in the same blocking solution and applied to the wells overnight at 4 °C. Following incubation, the cells were washed three times in PBS and then incubated with appropriate secondary antibodies (Additional file 1: Table S1) diluted in PBS for 1 h. Samples were washed in PBS, counterstained with 1 μg/ml Hoechst 33342 (Merck-Sigma) and mounted in FluoroMount‐G media (Thermo Fisher Scientific).

Human brain sections were first deparaffinized and then subjected to heat-induced epitope retrieval in sodium citrate (10 mM, pH = 6.0; Merck-Sigma) for 15 min. Mouse brain sections were permeabilized with PBS containing 0.5% Triton X‐100 (PBS-T) for 20 min. Both types of sections were blocked with 3% BSA in PBS-T for 1 h. Next, sections were incubated with primary antibodies (Additional file 1: Table S1) diluted in blocking solution overnight at 4 °C under slow nutation. After washing the sections three times in PBS, they were incubated with appropriate secondary antibodies (Additional file 1: Table S1) in PBS-T for 1 h at room temperature in the dark. After three further washing steps, samples were coverslipped with FluoroMount‐G mounting media. Nuclear counterstaining with Hoechst 33342 was performed on human samples for 5 min and on mouse samples for 10 min during the second washing step.

#### Fluorescence microscopy

Samples were examined using a Leica TCS SP5 laser scanning microscope (Leica Biosystems) with an HCX PL APO lambda blue 63 × /1.4 oil immersion objective or a VisiScope CSU-W1 spinning disk confocal microscope (Visitron Systems GmbH, Pulcheim, Germany) with a PlanApo N 60 × /1.42 oil immersion objective (both microscopes belonging to the Cellular Imaging Laboratory of the Biological Research Centre, Szeged, Hungary). Certain confocal and superresolution images were captured with a STEDYCON (Abberior Instruments, Göttingen, Germany) STED (stimulated emission depletion) superresolution system attached to an Axio Observer Z1 inverted epifluorescence microscope (Zeiss, Oberkochen, Germany) equipped with an alpha Plan-Apochromat 100 × /1.46 oil immersion objective. Multiple images were taken from approx. 5 µm depth of field in case of human cells and sections or 20 µm in case of mouse sections. These images were then merged into *z*-projections in FIJI. In some cases, the percentage of colocalization was provided to simplify the visualization of certain staining.

#### Quantification of astroglial localization of IL-1β

Mouse brain sections were stained for glial fibrillary acidic protein (GFAP) and IL-1β. *Z*-stack images (x: 222 μm; y: 222 μm; z: 20 μm; with 1 μm increments) were acquired with a VisiScope CSU-W1 confocal microscope at 60× magnification. Images were then processed with FIJI using a standardized protocol. Quantification was carried out on vehicle-treated and MCC950-treated animals 7 days after the injection of 4T1-tdT cells or sterile Krebs–Ringer solution. Pixel counts were determined for GFAP, IL-1β and IL-1β-GFAP colocalization and averaged over groups. Data were normalized to total pixel counts.

#### Determination of tumor size upon inflammasome inhibition

To assess the effect of inflammasome inhibition on the size of metastatic mammary tumors in the brain, intracardiac inoculation, a commonly used experimental model of brain metastasis, was adopted [35]. Two groups were created: the vehicle control and the MCC950-treated group. They received a single dose of intraperitoneal treatment daily between days 4 and 6 after tumor cell injection. After perfusion on day 7 postinjection, brains were prepared for sectioning. Coronal sections (30 μm thick) were used to measure the size of metastatic lesions in the neocortex.

#### Quantification of microgliosis

For the determination of peritumoral microgliosis with a quantitative approach, ionized calcium-binding adapter molecule 1 (Iba1)-stained coronal brain sections were used. The same tissue processing and microscopy protocol was employed for the determination of tumor size. We compared the average of Iba1-positive pixels and normalized Iba1-positive pixel counts to total pixel counts.

### RNA isolation and quantitative polymerase chain reaction (qPCR)

PCR experiments were carried out in cell cultures and tissue samples. In the in vitro setup, we cultured HAs in poly-l-lysine-coated 6-well plates. At a confluence of approximately 90%, HAs were treated with MDA-TGL CM or MDA-BrM2 CM in the presence or absence of 1 µM MCC950 for 24 h. Nontreated cells served as controls. Cells were collected in TRI Reagent (Thermo Fisher Scientific) for further processing. For ex vivo experiments, BALB/c mice were inoculated with 4T1-tdT cells and received treatments as described above. After 7 days, animals were transcardially perfused with PBS, and brains were dissected in a mouse brain matrix soaked in cold PBS. Olfactory bulbs and cerebella were cut off, and then cerebra were carefully halved along the longitudinal fissure; thus, we could separate the tumor-bearing side from the contralateral, control side. The hemispheres were separately homogenized in a Potter–Elvehjem homogenizer with a polytetrafluoroethylene pestle in 1 ml TRI Reagent. To avoid contamination, the homogenizer was thoroughly washed several times with distilled water between the samples.

Total RNA was isolated from TRI Reagent samples by using the Direct-zol RNA Miniprep Plus Kit (Zymed Laboratories, Irvine, CA, USA). To transcribe RNA to cDNA, a Maxima First Strand cDNA Synthesis Kit (Thermo Fisher Scientific) was used according to the manufacturer’s instructions. Amplification was performed using Luminaris HiGreen Master mix (Thermo Fisher Scientific) on a Bio-Rad CFX96 Real-Time thermocycler (Bio‐Rad, Hercules, CA, USA) under the following conditions: 40 cycles of 95 °C for 15 s, 60 °C for 30 s and 72 °C for 30 s, using the primers detailed in Additional file 1: Table S2.

### Protein extraction and western blot

Seven days after tumor cell injection, the brains of BALB/c mice from both the MCC950-treated and vehicle groups were processed similarly for RNA isolation but in 1 ml of cold homogenization buffer containing 10 mM PBS, SigmaFast protease inhibitor cocktail (Merck-Sigma) and 1% Triton X-100. Samples were then centrifuged (13 000×*g*, 20 min, 4 °C) to settle tissue debris, and the supernatant was used for further processing.

After measurement of protein concentration with the bicinchoninic acid assay (Thermo Fisher Scientific), we proceeded with protein precipitation using the methanol-chloroform method, as follows: (1) phase separation with equal part of 100% ice-cold methanol and 1/4 part of chloroform added to the homogenized samples and vortexing; (2) centrifugation (13 000×*g*, 5 min, 4 °C); (3) removal of aqueous phase, washing of the protein pellet with methanol and vortexing; (4) centrifugation (13 000×*g*, 5 min, 4 °C); and (5) removal of supernatant and drying of the pellet. Pellets were reconstituted in 2 × Laemmli buffer and heated to 95 °C for 5 min.

Equal amounts of proteins were resolved with standard denaturing sodium dodecyl sulfate‒polyacrylamide gel electrophoresis and transferred to polyvinylidene difluoride membranes (0.2 μm pore size; Bio-Rad). After blocking with 3% BSA in Tris-buffered saline containing 0.1% Tween-20 (TBS-T), membranes were incubated with primary antibodies (Additional file 1: Table S1) in TBS-T overnight at 4 °C. Blots were washed in TBS-T three times for 10 min and incubated for 1 h with appropriate horseradish peroxidase (HRP)-conjugated secondary antibodies (Additional file 1: Table S1). Proteins were visualized by chemiluminescence with the Clarity Chemiluminescence Substrate (Bio‐Rad) in a ChemiDoc MP System (Bio‐Rad). Band densities were quantified with Image Lab Software (version 5.2; Bio-Rad), and data were normalized to β-actin.

### Statistical analysis

All statistical tests were performed with GraphPad Prism (version 8.0.1.244; GraphPad Software, San Diego, CA, USA). A *P* value of less than 0.05 was considered significant. To determine the number of animals needed, power analysis was carried out with G* Power [14]. All experiments were performed in a blinded manner.

## Results

### NLRP3 inflammasome components are expressed in peritumoral astrocytes in TNBC brain metastases

**Fig. 1:**
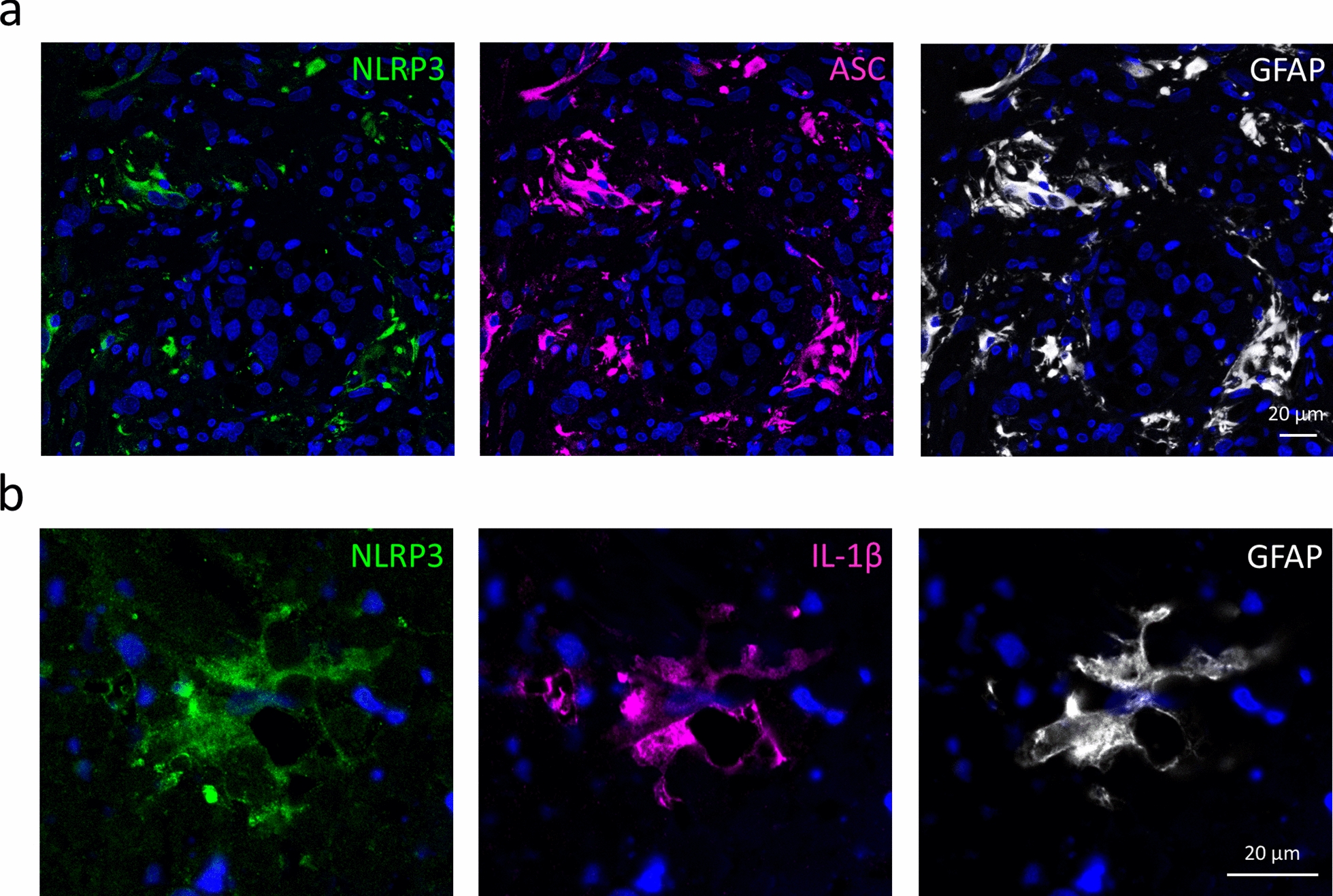
Expression of inflammasome components in peritumoral astrocytes in human TNBC brain metastases. **a** Representative immunofluorescence micrographs (maximum intensity projections of *z*-stacks) showing colocalization of NLRP3 and ASC with GFAP in advanced human TNBC brain metastases. A total of 95.3% of NLRP3-positive pixels and 98.8% of ASC-positive pixels colocalized with GFAP. **b** Representative immunofluorescence micrographs showing the colocalization of NLRP3 and IL-1β with GFAP in human TNBC brain metastases. Seventy-six percent of NLRP3-positive pixels and 74% of IL-1β-positive pixels colocalized with GFAP. *Blue: nuclei (Hoechst 33342 staining) in all figures*

To understand whether inflammasomes are primed and activated in brain metastases and to identify the cell type in which these phenomena arise, we first tested the expression of NLRP3 and ASC in human TNBC brain metastatic lesions. These inflammasome-associated proteins were highly and specifically expressed in peritumoral astrocytes, as shown by colocalization of NLRP3- and ASC-positive pixels with GFAP in the immunofluorescence images (Fig. 1a). As additional proof of inflammasome priming in astrocytes, human brain samples with breast cancer metastases were immunostained for IL-1β, which was also found to colocalize with GFAP and NLRP3 in the peritumoral regions (Fig. 1b). Inflammasome-related proteins could be detected neither in tumor cells nor in other stromal cells. In addition, astrocytes of control, healthy human brains were also devoid of detectable inflammasome-related signals (Additional file 1: Fig. S1).

**Fig. 2:**
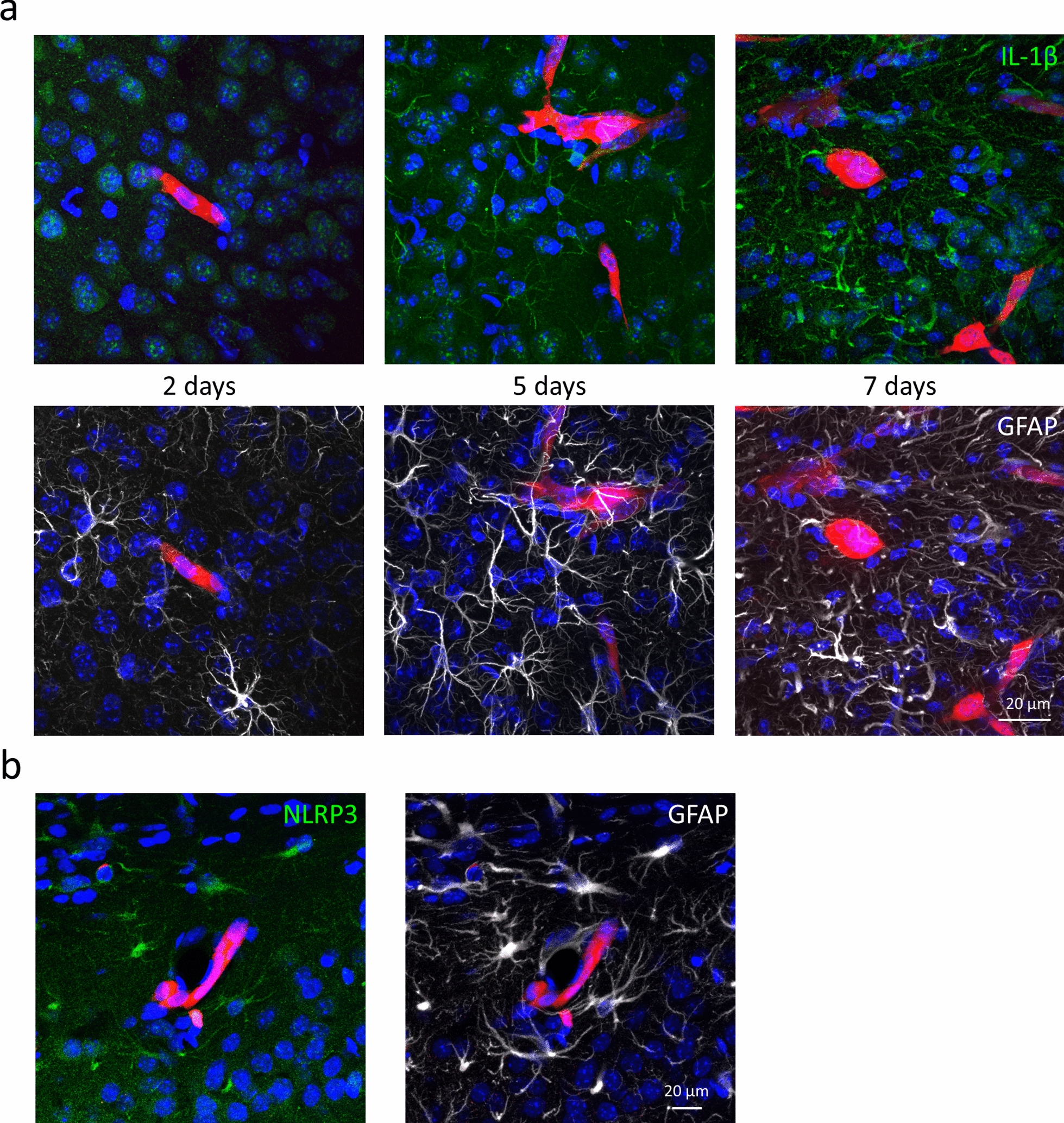
Upregulation of inflammasome components in peritumoral astrocytes in mouse TNBC brain metastases. **a** Representative immunofluorescence micrographs (maximum intensity projections of *z*-stacks) showing time-dependent upregulation of IL-1β expression and astrocyte reactivity in mouse TNBC brain metastases. At 5 and 7 days, 77% and 79% of cytoplasmic IL-1β-positive pixels colocalized with GFAP, respectively. At 5 and 7 days, 7% and 28% of GFAP-positive pixels were IL-1β-positive. **b** Representative maximum intensity projections of *z*-stacks showing expression of NLRP3 in peritumoral astrocytes 7 days after inoculation of the tumor cells. Ninety-five percent of NLRP3-positive pixels colocalized with GFAP. *Red: 4T1-tdT, in all figures*

As an experimental approach for understanding possible inflammasome activation in brain metastases, we injected 4T1-tdT cells into the right common carotid artery of mice. Two days after the inoculation of the tumor cells, no IL-1β was observed in the frontal, temporal and parietal cortical regions of the mice. At this stage, the majority of cancer cells are still stuck in the lumen of brain vessels [17]. After migration through the BBB (blood‒brain barrier), on day 5, IL-1β could already be seen in peritumoral astrocytes, while on day 7, the expression of IL-1β was even higher in GFAP-positive cells in the neighborhood of metastatic lesions (Fig. 2a). Peritumoral astrocyte reactivity also increased with time. The expression level of IL-1β correlated with the size of the metastatic lesion, being absent in tumor-free brain areas and more intense in the vicinity of large tumors in comparison to smaller ones (Additional file 1: Fig. S2). In parallel, NLRP3 was also upregulated in astroglia surrounding the tumors (Fig. 2b), similar to what we observed in the human triple-negative brain metastatic samples.

**Fig. 3:**
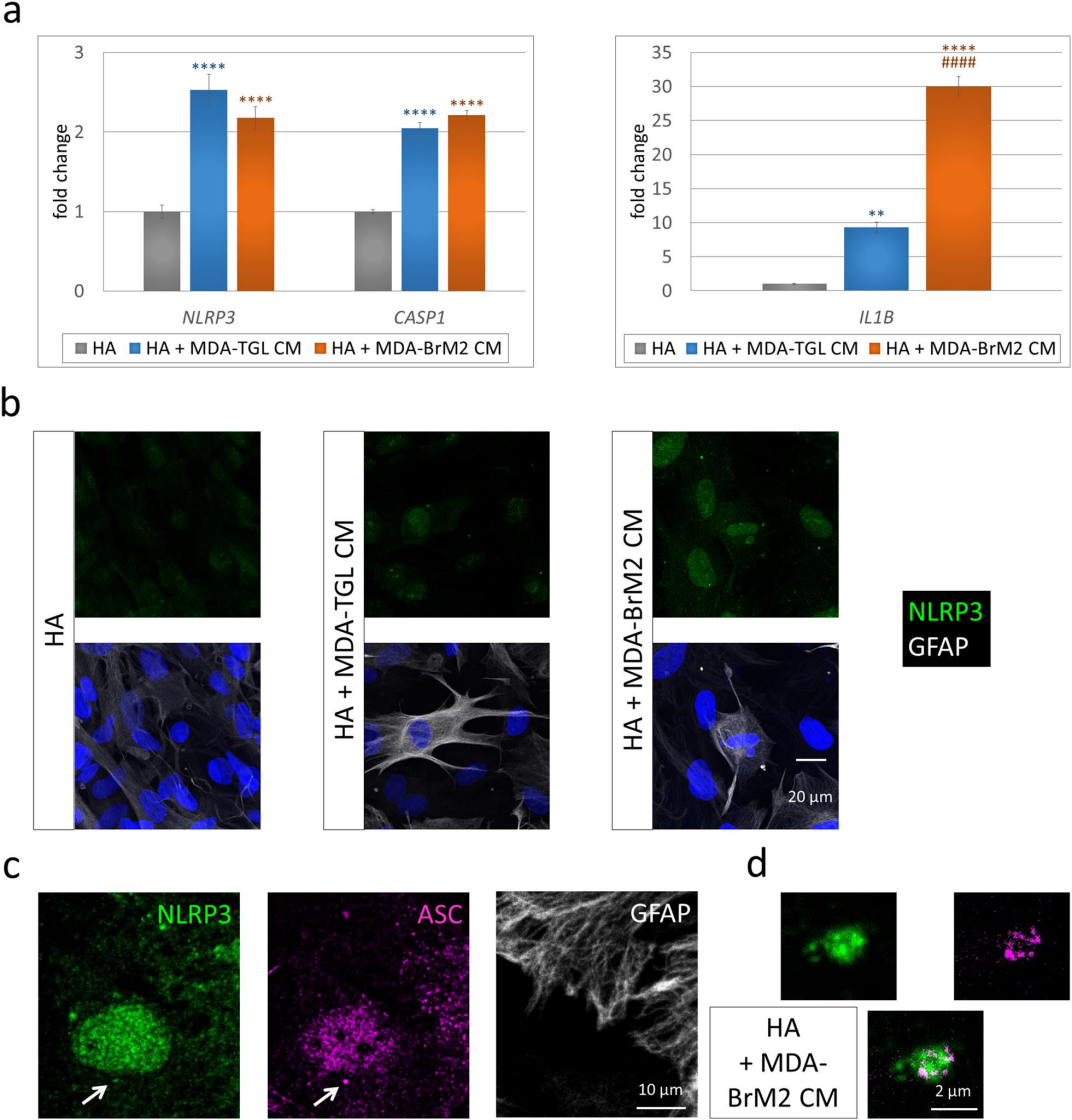
Priming and activation of the NLRP3 inflammasome in astrocytes exposed to breast cancer cell-secreted factors. **a** Upregulation of *NLRP3*, *CASP1* and *IL1B* gene expression in human astrocytes cultured for 24 h in human TNBC cell-conditioned medium. Graphs represent the fold change (normalized to *GAPDH*), average ± SEM (N = 3 independent experiments, each performed in triplicate). **P ≤ 0.01, ****P ≤ 0.0001 compared to control (HA), ^####^*P* ≤ 0.0001 compared to HA + MDA-TGL CM (ANOVA and Fisher’s LSD post hoc test). **b** Representative immunofluorescence micrographs (maximum intensity projections of *z*-stacks) showing upregulation of NLRP3 protein expression in human astrocytes cultured for 24 h in human TNBC cell-conditioned medium. **c** Representative immunofluorescence micrographs (maximum intensity projections of *z*-stacks) showing a perinuclear NLRP3- and ASC-positive speck-like structure (marked with an arrow) in a human astrocyte from a culture kept for 24 h in brain metastatic human TNBC cell-conditioned medium. **d** Magnification of the speck from image **c** ASC: STED image

To decipher the mechanisms, we performed in vitro experiments. First, we tested the priming of inflammasome components in astrocytes treated with breast cancer cell-secreted factors. Expression of *NLRP3* and *CASP1* genes showed a significant, more than twofold increase in human astrocytes exposed to culture media conditioned on parental (MDA-TGL) or brain metastatic (MDA-BrM2) TNBC cells compared to the control, nontreated astrocytes. Growth in the expression of *IL1B* was even more pronounced, being elevated almost tenfold in astrocytes exposed to the conditioned medium of parental breast cancer cells and approximately 30-fold in astrocytes cultured in the presence of factors secreted by brain metastatic cells (Fig. 3a). Second, we analyzed changes in inflammasome components at the protein level. Increased expression of NLRP3 protein was mainly detected in the nuclei of astrocytes treated with breast cancer cell-conditioned media (Fig. 3b). Nuclear localization is compatible with inflammasome activation [54]; however, as more direct proof of inflammasome assembly, NLRP3- and ASC-positive perinuclear speck-like structures were also observed (Fig. 3c, d). Using superresolution imaging, we observed that ASC formed the outer ring, whereas NLRP3 constituted the core of these structures (Fig. 3d), as suggested by previous studies [32].

### Inflammasome activation in astrocytes augments the proliferation of breast cancer cells in vitro

We hypothesized that inflammasome activation-induced IL-1β release in astrocytes might contribute to tumor cell proliferation. Indeed, administration of IL-1β to the cell cultures was able to promote proliferation of both human and mouse breast cancer cells. In both models, the number of breast cancer cells rose sharply from the third day on and reached a twofold difference in the case of the human cells and a threefold increase in mouse cells compared to the nontreated controls by the fourth day (Additional file 1: Fig. S3a, b).

Similarly, conditioned media collected from astrocytes fostered the replication of parental and brain metastatic MDA cells, as well as that of mouse 4T1 cells (Additional file 1: Figs. S4a, b, S5a, b). Especially in mouse cells, this was even stronger when conditioned media was collected from activated astrocytes, which had previously come in contact with tumor cell-derived factors. Here, the difference between the effect of naïve and activated astrocytes was approximately 1.75-fold (Additional file 1: Fig. S5b).

**Fig. 4:**
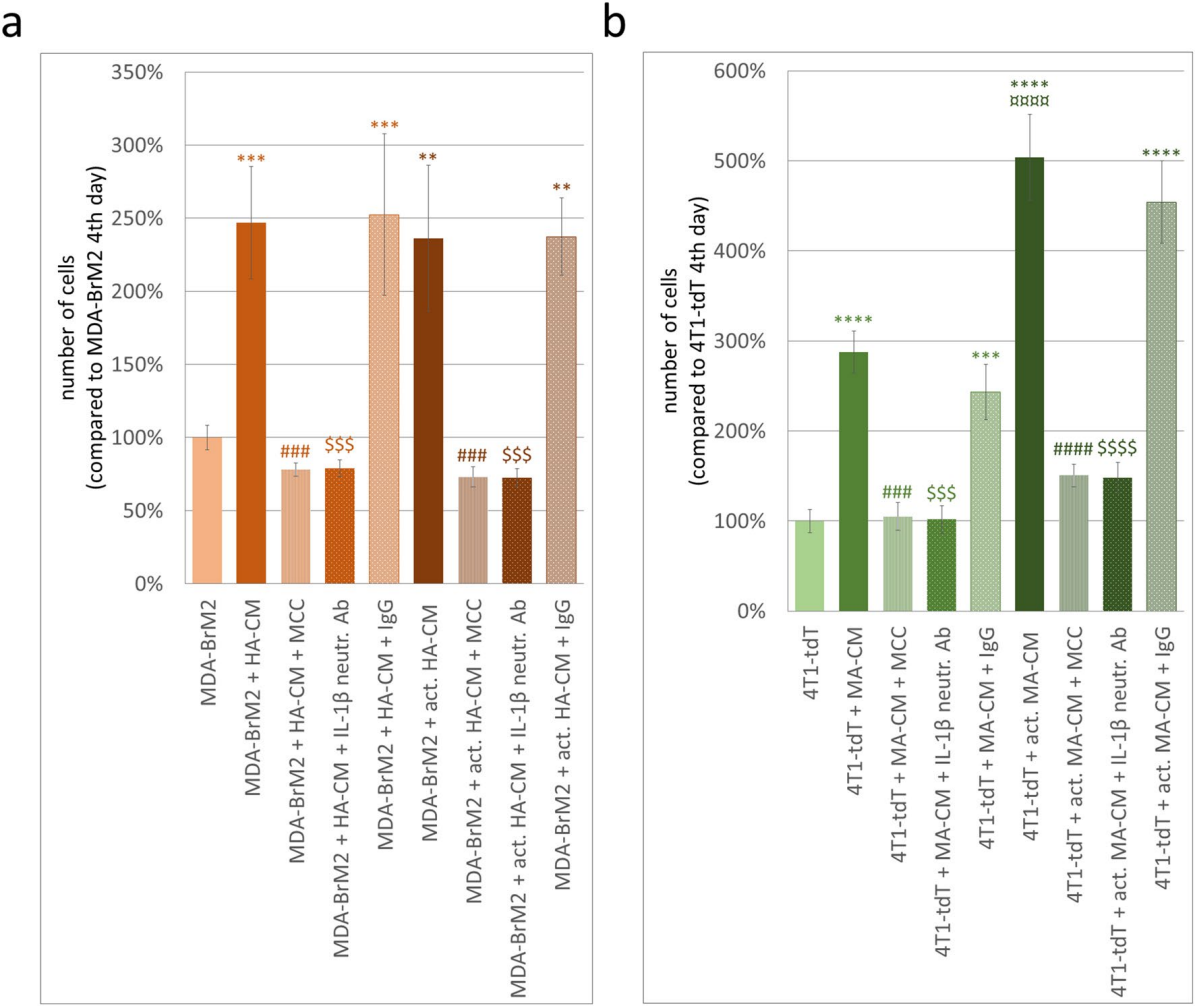
Proliferation of breast cancer cells in response to astrocyte-secreted IL-1β. **a** Proliferation of human brain metastatic breast cancer cells in media collected from human astrocytes or human astrocytes exposed to MCC950. IL-1β neutralizing or control IgG antibodies were added to astrocyte-conditioned medium before application to breast cancer cells. Graphs represent the percentage of cells on the 4th day after plating, average ± SEM (N = 2 independent experiments, each performed in duplicate, 5 different fields of view photographed from each well). **P ≤ 0.01, ***P ≤ 0.001 compared to control (MDA-BrM2), ^###^*P* ≤ 0.001 compared to “MDA-BrM2 + HA-CM” or “MDA-BrM2 + act. HA-CM”, ^$$$^*P* ≤ 0.001 compared to “MDA-BrM2 + HA-CM” and “MDA-BrM2 + HA-CM + IgG” or “MDA-BrM2 + act. HA-CM” and “MDA-BrM2 + act. HA-CM + IgG” (ANOVA and Fisher’s LSD post hoc test). **b** Proliferation of mouse breast cancer cells in media collected from mouse astrocytes or mouse astrocytes exposed to MCC950. IL-1β neutralizing or control IgG antibodies were added to astrocyte-conditioned medium before application to breast cancer cells. Graphs represent the percentage of cells on the 4th day after plating, average ± SEM (N = 2 independent experiments, each performed in duplicate, 5 different fields of view photographed from each well). ***P ≤ 0.001, ****P ≤ 0.0001 compared to control (4T1-tdT), ^###^*P* ≤ 0.001 compared to “4T1-tdT + MA-CM”, ^####^*P* ≤ 0.0001 compared to “4T1-tdT + act. MA-CM”, ^$$$^*P* ≤ 0.0001 compared to “4T1-tdT + MA-CM” and “4T1-tdT + MA-CM + IgG”, ^$$$$^*P* ≤ 0.0001 compared to “4T1-tdT + act. MA-CM” and “4T1-tdT + act. MA-CM + IgG”, ^¤¤¤¤^*P* ≤ 0.0001 compared to “4T1-tdT + MA-CM” (ANOVA and Fisher’s LSD post hoc test)

To prove that astrocyte-conditioned media increased the proliferation of breast cancer cells through inflammasome activation and IL-1β secretion, we treated astrocytes with MCC950, a specific NLRP3 inhibitor [9]. In addition, we neutralized secreted IL-1β in the astrocyte-conditioned media before adding it to breast cancer cells. First, we observed that MCC950 reversed breast cancer-induced increased expression of NLRP3 inflammasome-associated genes in astrocytes (Additional file 1: Fig. S6), probably through inhibition of IL-1β and a subsequent feedback effect on priming. More importantly, both MCC950 and neutralization of IL-1β prevented astrocyte-conditioned media-induced augmentation of breast cancer cell proliferation in vitro, both in the human and mouse models (Fig. 4a, b). The number of tumor cells cultured in the medium of naïve or activated astrocytes treated with MCC950 was highly similar to the number of control cells. Similarly, neutralization of IL-1β almost completely prevented increased proliferation of breast cancer cells coming in contact with the culture media of astrocytes.

These results clearly prove that inflammasome activation in astrocytes induces IL-1β secretion, which in turn facilitates the proliferation of breast cancer cells.

### Inhibition of the NLRP3 inflammasome arrests metastatic growth in the brain

**Fig. 5:**
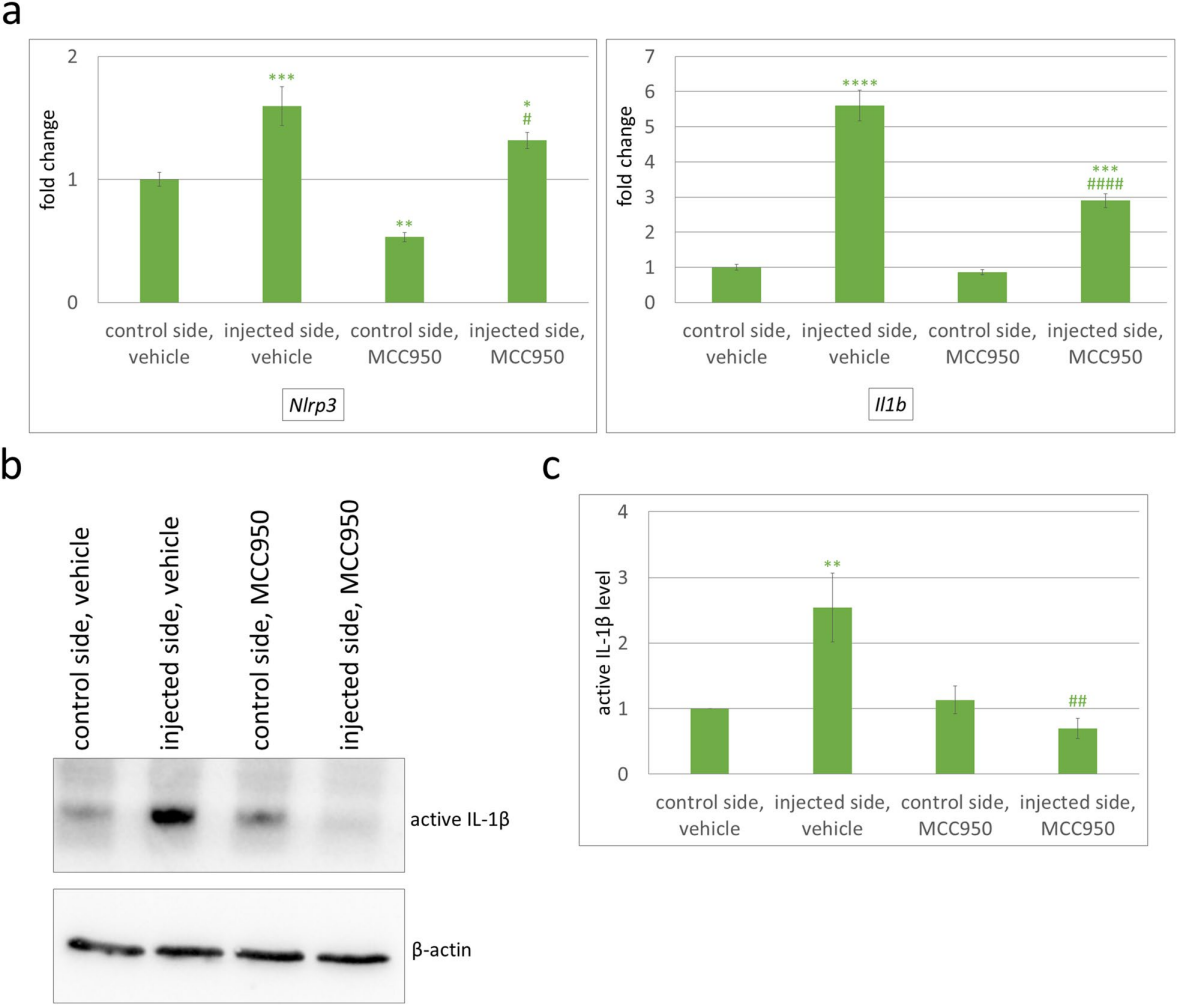
Priming and activation of the NLRP3 inflammasome in the brains of mice bearing TNBC cells. **a** Expression of the *Nlrp3* and *Il1b* genes in the brains of mice 7 days after intracarotid injection of 4T1-tdT cells treated with vehicle (DMSO in PBS) or 10 mg/kg MCC950. Graphs represent the fold change (normalized to *GAPDH*), average ± SEM (N = 3 independent experiments, each performed in triplicate). *P ≤ 0.05, **P ≤ 0.01, ***P ≤ 0.001, ****P ≤ 0.0001 compared to “control side, vehicle”, ^#^*P* ≤ 0.05, ^####^*P* ≤ 0.0001 compared to “injected side, vehicle”, respectively (ANOVA and Fisher’s LSD post hoc test). **b** Representative western blot showing the expression of active IL-1β in the respective hemispheres. **c** Quantitative analysis of active IL-1β levels compared to β-actin expression, as assessed by western blot. Graphs represent the average ± SEM (N = 3 independent experiments). **P ≤ 0.01 compared to “control side, vehicle”, ^##^*P* ≤ 0.01 compared to “injected side, vehicle”, respectively (ANOVA and Fisher’s LSD post hoc test)

As a next step, we aimed to understand the effects of NLRP3 activation and its pharmacological inhibition in our in vivo brain metastasis model. Animals were administered MCC950, which is able to effectively cross the BBB [37], during and after the assumed extravasation of the tumor cells into the brain (Additional file 1: Fig. S7a). As expected from the immunofluorescence studies, breast cancer brain metastases were associated with the upregulation of *Nlrp3* and *Il1b* gene expression in the hemisphere comprising the metastatic cells compared to the contralateral side, serving as an internal control. However, MCC950 significantly reversed the tumor-induced upregulation of *Nlrp3* and *Il1b* gene expression (Fig. 5a).

Since the precursor and active forms of IL-1β cannot be distinguished by immunofluorescence, we performed western blot studies, where the mature cytokine can be recognized by its molecular weight (17 kDa). In the tumor-containing hemispheres, we detected high levels of active IL-1β as a readout for inflammasome activation. The NLRP3 inhibitor reduced the amount of active IL-1β to that observed in the control (tumor-free) side (Fig. 5b, c).

**Fig. 6:**
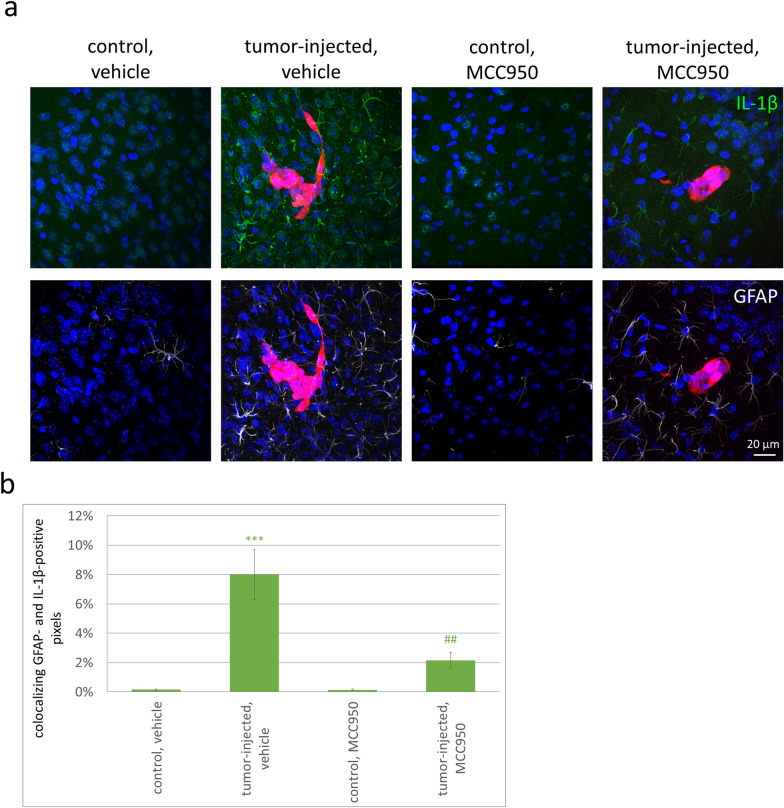
NLRP3 inhibition-induced protection against IL-1β upregulation in mouse breast cancer brain metastasis.** a** Representative immunofluorescence micrographs (maximum intensity projections of *z*-stacks) showing the expression of IL-1β and GFAP in the brains of mice inoculated with 4T1-tdT TNBC cells (tumor-injected) or Krebs–Ringer solution (control) and treated with vehicle (DMSO in PBS) or 10 mg/kg MCC950 7 days after the injection of the tumor cells. **b** Quantitative analysis of colocalizing GFAP- and IL-1β-positive pixels compared to all pixels. Graphs represent the average ± SEM (N = 3 independent experiments, n = 5 sections/animal, 8 ROIs/section). ***P ≤ 0. 01 compared to “control, vehicle”, ^##^*P* ≤ 0.01 compared to “tumor-injected, vehicle”, respectively (ANOVA and Fisher’s LSD post hoc test). *ROI* = *region of interest*

Next, we assessed the effect of MCC950 on astrocytic IL-1β expression. The colocalization of GFAP- and IL-1β-positive signals was high in the tumor-bearing animals, while no detectable IL-1β was observed in the control mice. In addition, NLRP3 inhibition proved to be protective against IL-1β upregulation in peritumoral astrocytes in the mouse breast cancer brain metastasis model, resulting in an approximately 75% decrease in the percentage of IL-1β- and GFAP-double-positive pixels (Fig. 6a, b). The reduction in astrocyte reactivity was mild (Additional file 1: Fig. S7b), while reversal of microgliosis was significant in response to MCC950 in mice bearing breast cancer metastases in their brains (Additional file 1: Fig. S7c and d).

**Fig. 7:**
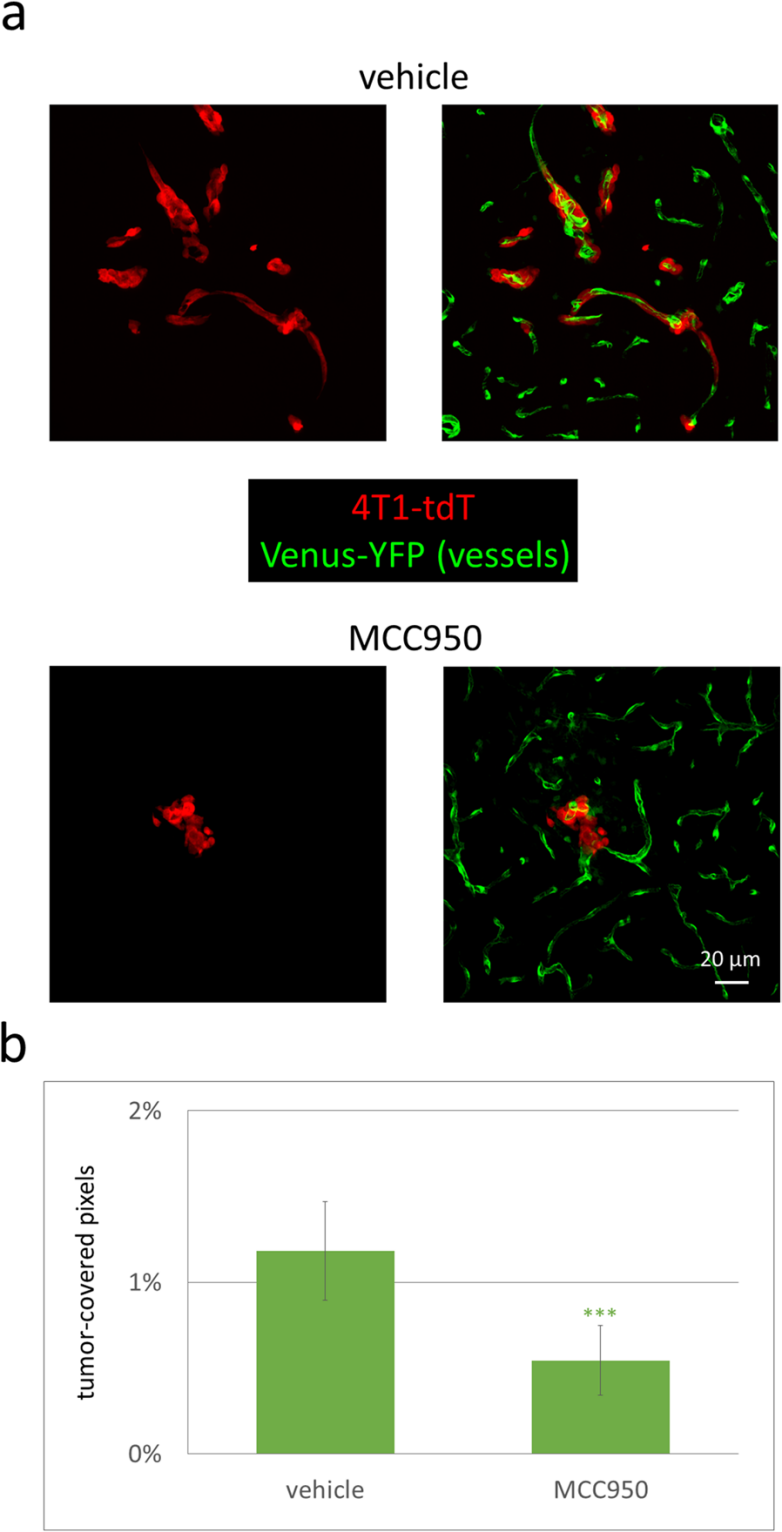
Retarded growth of metastatic tumors in the brains of mice treated with MCC950. **a** Representative confocal micrographs (maximum intensity projections of *z*-stacks) showing 4T1-tdT tumors in mice treated with vehicle (DMSO in PBS) or 10 mg/kg MCC950 7 days after the injection of the tumor cells. **b** Quantitative analysis of tumor-covered pixels compared to all pixels. Graphs represent the average ± SEM (N = 3 independent experiments, n = 5 sections/animal, 16 ROIs/5 sections). *** *P* ≤ 0.001 (unpaired Student’s *t* test)

Most importantly, systemic administration of MCC950 retarded the perivascular growth of breast tumors in the brains of the mice. Tumor-covered pixels were reduced to less than 50% in mice receiving MCC950 in comparison to those treated with vehicle (Fig. 7a, b).

These data suggest that inhibition of the NLRP3 inflammasome decreases not only inflammasome-associated gene expression but also the level of active IL-1β originating from peritumoral astrocytes and, most importantly, tumor growth.

## Discussion

Overcoming the BBB, the tightest endothelial barrier in the organism [57], is only the first stage of brain metastasis development. After crossing the vessel wall, as a second phase, tumor cells have to survive in the brain environment, and both steps may be facilitated by proinflammatory cytokines and chemokines [13].

Following migration through the cerebral endothelium [18], cancer cells pass through the glia limitans perivascularis formed by astrocytic endfeet; however, later, astrocytes are expelled onto the surface of the growing tumor [17]. Metastatic cells proliferate in the perivascular niche and coopt the vessels of the brain, yet interaction with astrocytes remains crucial in determining the fate of the tumors. Since astrocytes may be both “friends or foes” of invading cells, these latter must first defeat the antitumor mechanisms initiated by astroglia to take advantage of their prometastatic features [56]. Inflammation may also have both pro- and antitumoral effects [65]. Although glia-tumor interactions have been rather well characterized, the role of astrocytes in the formation of an inflammatory metastatic environment remains largely elusive [10]. In an in vitro model, astrocytes were shown to secrete inflammatory cytokines (IL-6, tumor necrosis factor-α (TNF-α) and IL-1β) in the presence of lung cancer cells, resulting in increased proliferation of the tumor cells [46]. In vivo, the release of interferon-α (IFN-α) and TNF-α has been shown in astrocytes forming gap junctions with lung and breast carcinoma cells, supporting tumor growth [6]. Nevertheless, to our knowledge, the role of inflammasomes in brain metastasis formation has remained completely unknown thus far. By using in vitro and in vivo models, as well as human brain metastatic tissue, we show that through NLRP3 inflammasome activation, astroglia are the main players in the formation of an inflammatory environment in TNBC brain metastases. We observed that the NLRP3 inflammasome is primed and activated in peritumoral astrocytes. As a consequence, IL-1β is secreted in the tumor environment, which in turn enhances the proliferation of metastatic cells.

According to our observations, IL-1β expression was restricted to peritumoral astrocytes, while in gliomas, both the host cells and the tumor cells were shown to take part in inflammasome signaling. Particularly, high-grade glioma-associated microglia were observed to secrete IL-1β via the NLRP1 inflammasome, promoting tumor progression [28]. In addition, in glioma cells, overexpression of NLRP12 and NLRC4 inflammasome-associated genes and proteins contributed to malignancy and poor prognosis [42]. In a lung brain metastasis xenograft model, *Nlrp3* and *Il1b* transcripts were upregulated in tumor-associated macrophages, suggesting possible inflammasome activation [44].

Previously, brain metastatic breast cancer cells were shown to secrete IL-1β in vitro, which activated Notch and transforming growth factor-β_2_ (TGF-β_2_) signaling in astrocytes [15, 59]. Moreover, inflammasome activation in breast cancer cells was found to promote their invasiveness and metastasis formation [55]. However, in our animal model and in human brain metastatic tissue, no IL-1β expression was detected in tumor cells.

IL-1β was almost undetectable in the neocortex until the tumor cells migrated through the BBB, while its expression increased in peritumoral astrocytes in a time- and tumor size-dependent manner. As another proof of bidirectional communication between astrocytes and tumor cells, in the in vitro mouse model, the culture media collected from astrocytes previously coming in contact with the soluble factors released by breast cancer cells induced a more intense proliferation of the tumor cells than media of naïve astrocytes. Although cancer cell-derived factors leading to astrocytic NLRP3 inflammasome activation have not been identified, several molecules might be responsible for this phenomenon, including released TGF-β, soluble CD44, extracellular mitochondrial DNA or ATP from dying tumor cells [21]. In primary breast tumors, both in mouse and human carcinomas, NLRP3 activation was primarily observed in host cells, specifically in cancer-associated fibroblasts (CAFs). Activation of NLRP3 signaling in CAFs facilitated tumor growth and metastasis by modulating immune and endothelial cells [12].

In our study, astrocyte-secreted IL-1β increased the proliferation of breast cancer cells both in vitro and in vivo, while inhibition of NLRP3 activation in astrocytes or neutralization of secreted IL-1β prevented astrocyte-induced increased proliferation of TNBC cells in both mouse and human models. Although IL-1β might also be secreted in an inflammasome-independent manner [39], the comparable effect of NLRP3 inflammasome inhibition using MCC950 and IL-1β neutralization using specific antibodies suggests the involvement of inflammasomes in this process. Since MCC950, a specific NLRP3 inflammasome inhibitor, reduced astrocyte-enhanced tumor cell proliferation to the control level, the role of other inflammasome types can also be excluded.

Most importantly, by administration of MCC950, we reduced the growth of TNBC metastases in the brains of the mice. MCC950 was the first small molecule to block the NLRP3 inflammasome very potently and selectively [9] by directly targeting the ATP-hydrolysis motif of NLRP3 [8]. Since NLRP3 inflammasome activation has been linked to a series of diseases in the CNS and the periphery, several NLRP3 inhibitors have been developed in the last few years, some of them advancing to clinical trials [7]. Inflammasome inhibitors are considered promising therapeutic agents against different types of cancer as well [60]. Although potential liver toxicity issues hindered MCC950 from continuing to phase 2 [38], the ability of this and similar small molecules to readily cross the BBB is of great advantage since the BBB is the most important obstacle in front of the brain uptake of most pharmaceuticals [25]. Currently, several companies are designing and testing novel NLRP3 inhibitors, both MCC950 analogs and structurally unrelated compounds, to find potent, selective and safe drugs for the treatment of inflammatory diseases [11].

Using an NLRP3 inhibitor with low translational potential is only one limitation of our study. The single mouse TNBC cell line and the two, MDA-derived human cell lines represent models void of the heterogeneity of patient-derived xenografts. However, both 4T1 and MDA-BrM2 are very well-characterized brain metastatic cells [1, 47].

## Conclusions

Taken together, to our knowledge, we are the first to show that peritumoral reactive astrocytes promote the proliferation of TNBC cells in the brain through NLRP3 inflammasome-dependent secretion of IL-1β. Brain metastases are among the most aggressive and the least curable malignant tumors; therefore, we need novel therapies targeting mechanisms that contribute to the proliferation of metastatic cells in the brain. Based on our results, the inflammatory brain metastatic environment, especially peritumoral reactive astrocytes, could be the focus of future therapies. We suggest that inflammasome inhibition might become a therapeutic option in this currently incurable disease.

### Supplementary Information

**Additional file 1: Table S1.** Antibodies used in the experiments. **Table S2.** Primers used for PCR. **Fig. S1.** Absence of detectable NLRP3 and IL-1β expression in astrocytes of the healthy human brain. Representative immunofluorescence micrographs (maximum intensity projections of *z*-stacks) showing the absence of NLRP3 and IL-1β staining in GFAP-positive cells in healthy human brain sections. **Fig. S2.** Upregulation of IL-1β in association with TNBC metastases in the mouse brain. Representative immunofluorescence micrographs (maximum intensity projections of *z*-stacks) showing tumor size-dependent upregulation of IL-1β expression in mouse TNBC brain metastases 7 days after inoculation of the tumor cells. **Fig. S3.** Proliferation of breast cancer cells in response to IL-1β. **a** Proliferation of human breast cancer cells in response to 10 ng/ml IL-1β. Graphs represent the average ± SEM (N = 3 independent experiments, each performed in duplicate, 5 different fields of view photographed from each well). *P ≤ 0.05, **P ≤ 0.01, ****P ≤ 0.0001 compared to control (MDA-TGL or MDA-BrM2, respectively) (ANOVA and Fisher’s LSD post hoc test). **b** Proliferation of mouse breast cancer cells in response to 10 ng/ml IL-1β. Graphs represent the average ± SEM (N = 3 independent experiments, each performed in duplicate, 5 different fields of view photographed from each well). **P ≤ 0.01, ****P ≤ 0.0001 compared to control (4T1-tdT) (ANOVA and Fisher’s LSD post hoc test). **Fig. S4.** Proliferation of breast cancer cells in response to astrocyte-conditioned media. **a** Representative phase contrast images showing the proliferation of human breast cancer cells cultured in human astrocyte-conditioned media. **b** Representative phase contrast images showing the proliferation of mouse breast cancer cells cultured in mouse astrocyte-conditioned media. Quantitative analyses are shown in Additional file 1: Fig. S5. **Fig. S5.** Proliferation of breast cancer cells in response to astrocyte-secreted factors (quantitative data). **a** Proliferation of human breast cancer cells cultured in human astrocyte-conditioned media. Graphs represent the average ± SEM (N = 3 independent experiments, each performed in duplicate, 5 different fields of view photographed from each well). **P ≤ 0.01, ****P ≤ 0.0001 compared to the same day’s control (MDA-TGL or MDA-BrM2, respectively) (ANOVA and Fisher’s LSD post hoc test). **b** Proliferation of mouse breast cancer cells cultured in mouse astrocyte-conditioned media. Graphs represent the average ± SEM (N = 3 independent experiments, each performed in duplicate, 5 different fields of view photographed from each well). *P ≤ 0.05, **P ≤ 0.01, ***P ≤ 0.001, ****P ≤ 0.0001 compared to the same day’s control (4T1-tdT), ^####^*P* ≤ 0.0001 compared to the same day’s “4T1-tdT + act. MA-CM” (ANOVA and Fisher’s LSD post hoc test). **Fig. S6.** NLRP3 inhibition-induced changes in the expression of NLRP3 inflammasome-associated genes in astrocytes. Expression of *NLRP3* and *IL1B* genes in human astrocytes cultured for 24 h in human TNBC cell-conditioned medium in the presence or absence of 1 μM MCC950. Graphs represent the fold change (normalized to *GAPDH*), average ± SEM (N = 3 independent experiments, each performed in triplicate). **P ≤ 0.01, ***P ≤ 0.001, ****P ≤ 0.0001 compared to control (HA), ^#^*P* ≤ 0.05, ^####^*P* ≤ 0.0001 compared to HA + MDA-TGL CM or HA + BrM2 CM, respectively (ANOVA and Fisher’s LSD post hoc test). **Fig. S7.** NLRP3 inhibition-induced reduction in gliosis associated with brain metastasis. **a** Schematic representation of the in vivo model (details presented in the “Materials and methods” section). **b** Percentage of GFAP-positive pixels compared to all pixels in brain sections of mice inoculated with 4T1-tdT TNBC cells (tumor-injected) or Krebs–Ringer solution (control) and treated with vehicle (DMSO in PBS) or 10 mg/kg MCC950 7 days after the injection of the tumor cells. Graphs represent the average ± SEM (N = 3 independent experiments, n = 8 sections/animal). **P ≤ 0.01 compared to “control, vehicle” (ANOVA and Fisher’s LSD post hoc test). **c** Representative immunofluorescence micrographs (maximum intensity projections of *z*-stacks) showing a reduction in peritumoral Iba1 expression in response to systemic MCC950 treatment in mouse TNBC brain metastases 7 days after inoculation of the tumor cells. **d** Ratio of Iba1-positive pixels in MCC950-treated animals compared to all pixels. Graphs represent the average ± SEM (N = 3 independent experiments, n = 6 sections/animal, 8 ROIs/6 sections). **P ≤ 0.01 (unpaired Student’s *t* test).

## Data Availability

The data that support the findings of this study are available from the corresponding authors upon reasonable request.

## Abbreviations

4T1-tdT: 4T1-tdTomato cell line
act. HA-CM: Activated human astrocyte-conditioned medium
act. MA-CM: Activated mouse astrocyte-conditioned medium
ASC: Apoptosis-associated speck-like protein containing a caspase activation and recruitment domain
BBB: Blood‒brain barrier
BSA: Bovine serum albumin
CAFs: Cancer-associated fibroblasts
CNS: Central nervous system
DMEM: Dulbecco’s modified Eagle’s medium
DMSO: Dimethyl sulfoxide
FBS: Fetal bovine serum
FIJI: Fiji is just ImageJ
GFAP: Glial fibrillary acidic protein
HA-CM: Human astrocyte-conditioned medium
HAs: Human astrocytes
HRP: Horseradish peroxidase
Iba1: Ionized calcium-binding adapter molecule 1
IFN-α: Interferon-α
IL-1β: Interleukin-1β
L1-CAM: L1 cell adhesion molecule
MA-CM: Mouse astrocyte-conditioned medium
MAs: Mouse astrocytes
MDA-BrM2: MDA-MB-231-BrM2 (brain metastatic) cell line
MDA-TGL: MDA-231-HSV1-TK/GFP/Fluc (parental) cell line
NLRP3: Nucleotide-binding and oligomerization domain, leucine rich repeat and pyrin domain-containing protein 3
PBS: Phosphate buffered saline
PBS-T: PBS containing 0.5% Triton X‐100
PFA: Paraformaldehyde
qPCR: Quantitative polymerase chain reaction
RPMI: Roswell Park Memorial Institute
STED: Stimulated emission depletion
TBS-T: Tris-buffered saline containing 0.1% Tween-20
TGF-β_2_: Transforming growth factor-β_2_
TNBC: Triple-negative breast cancer
TNF-α: Tumor necrosis factor-α
Venus mouse: FVB/Ant:TgCAG‐yfp_sb #27, YFP-expressing transgenic mouse strain
