# Supplementary material for: Inflammasome activation in peritumoral astrocytes is a key player in breast cancer brain metastasis development
Source: Acta Neuropathol Commun. 2023 Sep 25;11:155. doi: 10.1186/s40478-023-01646-2 (PMC10521486; doi:10.1186/s40478-023-01646-2)
Supplement: Supplementary file 1 — Additional file 1: Table S1. Antibodies used in the experiments. Table S2. Primers used for PCR. Fig. S1. Absence of detectable NLRP3 and IL-1β expression in astrocytes of the healthy human brain. Representative immunofluorescence micrographs (maximum intensity projections of z-stacks) showing the absence of NLRP3 and IL-1β staining in GFAP-positive cells in healthy human brain sections. Fig. S2. Upregulation of IL-1β in association with TNBC metastases in the mouse brain. Representative immunofluorescence micrographs (maximum intensity projections of z-stacks) showing tumor size-dependent upregulation of IL-1β expression in mouse TNBC brain metastases 7 days after inoculation of the tumor cells. Fig. S3. Proliferation of breast cancer cells in response to IL-1β. a Proliferation of human breast cancer cells in response to 10 ng/ml IL-1β. Graphs represent the average ± SEM (N = 3 independent experiments, each performed in duplicate, 5 different fields of view photographed from each well). *P ≤ 0.05, **P ≤ 0.01, ****P ≤ 0.0001 compared to control (MDA-TGL or MDA-BrM2, respectively) (ANOVA and Fisher’s LSD post hoc test). b Proliferation of mouse breast cancer cells in response to 10 ng/ml IL-1β. Graphs represent the average ± SEM (N = 3 independent experiments, each performed in duplicate, 5 different fields of view photographed from each well). **P ≤ 0.01, ****P ≤ 0.0001 compared to control (4T1-tdT) (ANOVA and Fisher’s LSD post hoc test). Fig. S4. Proliferation of breast cancer cells in response to astrocyte-conditioned media. a Representative phase contrast images showing the proliferation of human breast cancer cells cultured in human astrocyte-conditioned media. b Representative phase contrast images showing the proliferation of mouse breast cancer cells cultured in mouse astrocyte-conditioned media. Quantitative analyses are shown in Additional file 1: Fig. S5. Fig. S5. Proliferation of breast cancer cells in response to astrocyte-secreted factors (quantitative [file 40478_2023_1646_MOESM1_ESM.docx]

# Additional tables and figures

**Table S1. Antibodies used in the experiments.**

| **Method** | **Antibody** | **Host** | **Dilution** | **Source** | **Cat. number** |
| --- | --- | --- | --- | --- | --- |
| IF | anti-NLRP3 | goat | 1:100 | Bio-Techne, Minneapolis, MN, USA | NBP2-76806 |
| IF | anti-GFAP | rabbit | 1:300 | Abcam, Cambridge, UK | ab7260 |
| IF | anti-GFAP | mouse | 1:200 | Sigma-Aldrich | G3893 |
| IF | anti-ASC | mouse | 1:100 | Santa Cruz Biotech., Santa Cruz, CA, USA | sc-271054 |
| IF | anti-IL1β | rabbit | 1:200 | Abcam | ab9722 |
| IF | anti-Iba1 | goat | 1:200 | Abcam | ab5076 |
| IF | anti-mouse IgG, Alexa Fluor Plus 647 | donkey | 1:500 | Thermo F. Scientific | A32787 |
| IF | anti-rabbit IgG, Alexa Fluor Plus 488 | donkey | 1:500 | Thermo F. Scientific | A32790 |
| IF | anti-goat IgG, Alexa Fluor 594 | donkey | 1:500 | Thermo F. Scientific | A-11058 |
| IF | anti-goat IgG, Alexa Fluor A488 | donkey | 1:500 | Thermo F. Scientific | A-11055 |
| IF | anti-rabbit IgG, Alexa Fluor Plus 594 | donkey | 1:500 | Thermo F. Scientific | A32754 |
| IF | anti-rabbit IgG, Alexa Fluor Plus 647 | donkey | 1:500 | Thermo F. Scientific | A32795 |
| IF | anti-goat IgG, Alexa Fluor Plus 647 | donkey | 1:500 | Thermo F. Scientific | A32849 |
| IF | anti-mouse IgG, Alexa Fluor 488 | goat | 1:500 | Thermo F. Scientific | A-11029 |
| IF | anti-rabbit IgG, Alexa Fluor 647 | goat | 1:500 | Thermo F. Scientific | A-21245 |
| WB | anti-IL1β | rabbit | 1:1000 | Abcam | ab9722 |
| WB | anti-β-actin | mouse | 1:10000 | Sigma-Aldrich | A5441 |
| WB | anti-rabbit IgG, HRP | goat | 1:3000 | Jackson ImmunoRes., West Grove, PN, USA | 111-035-003 |
| WB | anti-mouse IgG, HRP | goat | 1:3000 | Thermo F. Scientific | G21040 |
| neutr. | anti-IL1β (human) | goat | 1:150 (2 µg/1.5 ml) | Bio-Techne | AF-201-NA |
| neutr. | anti-IL1β (mouse) | goat | 1:150 (2 µg/1.5 ml) | Bio-Techne | AF-401-NA |
| neutr. | IgG | goat | 1:750 (2 µg/1.5 ml) | Bio-Techne | AB-108-C |

IF = immunofluorescence, WB = western blot, neutr. = neutralization, HRP = horseradish peroxidase, IgG = immunoglobulin G

**Table S2. Primers used for PCR.**

| **Gene** | **Forward primer (5' → 3')** | **Reverse primer (5' → 3')** |
| --- | --- | --- |
| *NLRP3* (hu) | GTGCCGTGTTCACTGCCTGGTAT | AGTGGGATTCGAAACACGTGCA |
| *CASP1* (hu) | TCTTGGAGACATCCCACAATGGGC | TGCGCTCTACCATCTGGCTGC |
| *IL1B* (hu) | AGCTCGCCAGTGAAATGATG | GCCCTTGCTGTAGTGGTGGT |
| *Nlrp3* (m) | GGCGAGACCTCTGGGAAAAA | CTTCAAGGCTGTCCTCCTGG |
| *Il1b* (m) | TGCCACCTTTTGACAGTGATG | TGATGTGCTGCTGCGAGATT |
| *GAPDH* | GTGAAGGTCGGTGTCAACG | GTGAAGACGCCAGTAGACTC |

hu = human, m = mouse


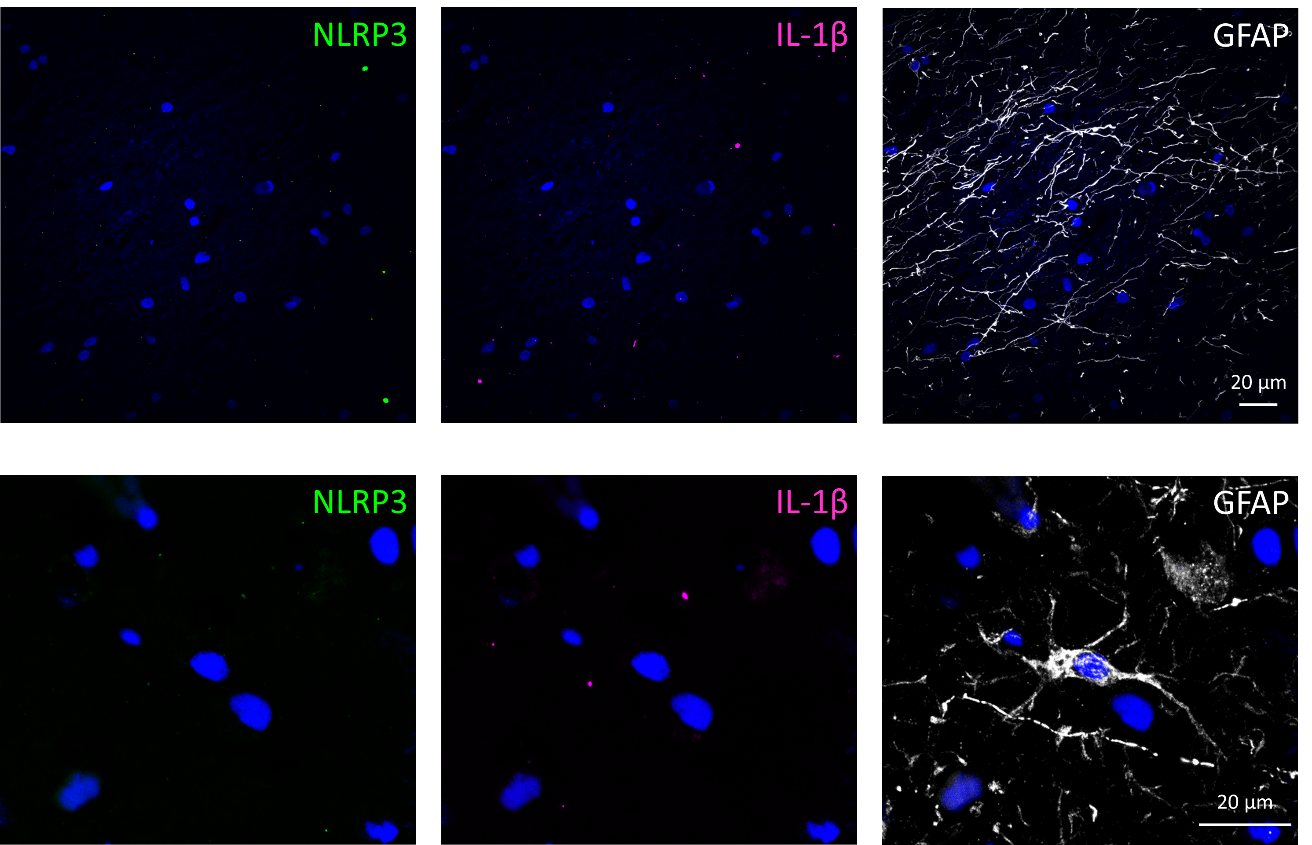


**Fig. S1. Absence of detectable NLRP3 and IL-1β expression in astrocytes of the healthy human brain.** Representative immunofluorescence micrographs (maximum intensity projections of *z*-stacks) showing the absence of NLRP3 and IL-1β staining in GFAP-positive cells in healthy human brain sections.


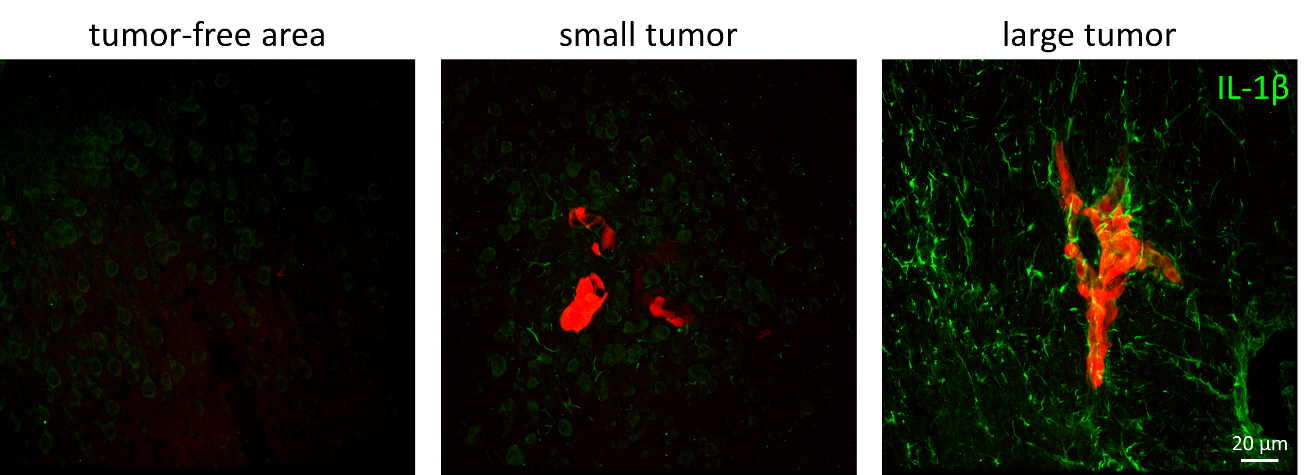


**Fig. S2. Upregulation of IL-1β in association with triple negative breast cancer metastases in the mouse brain.** Representative immunofluorescence micrographs (maximum intensity projections of *z*-stacks) showing tumor size-dependent upregulation of IL-1β expression in mouse triple negative breast cancer brain metastases 7 days after inoculation of the tumor cells.


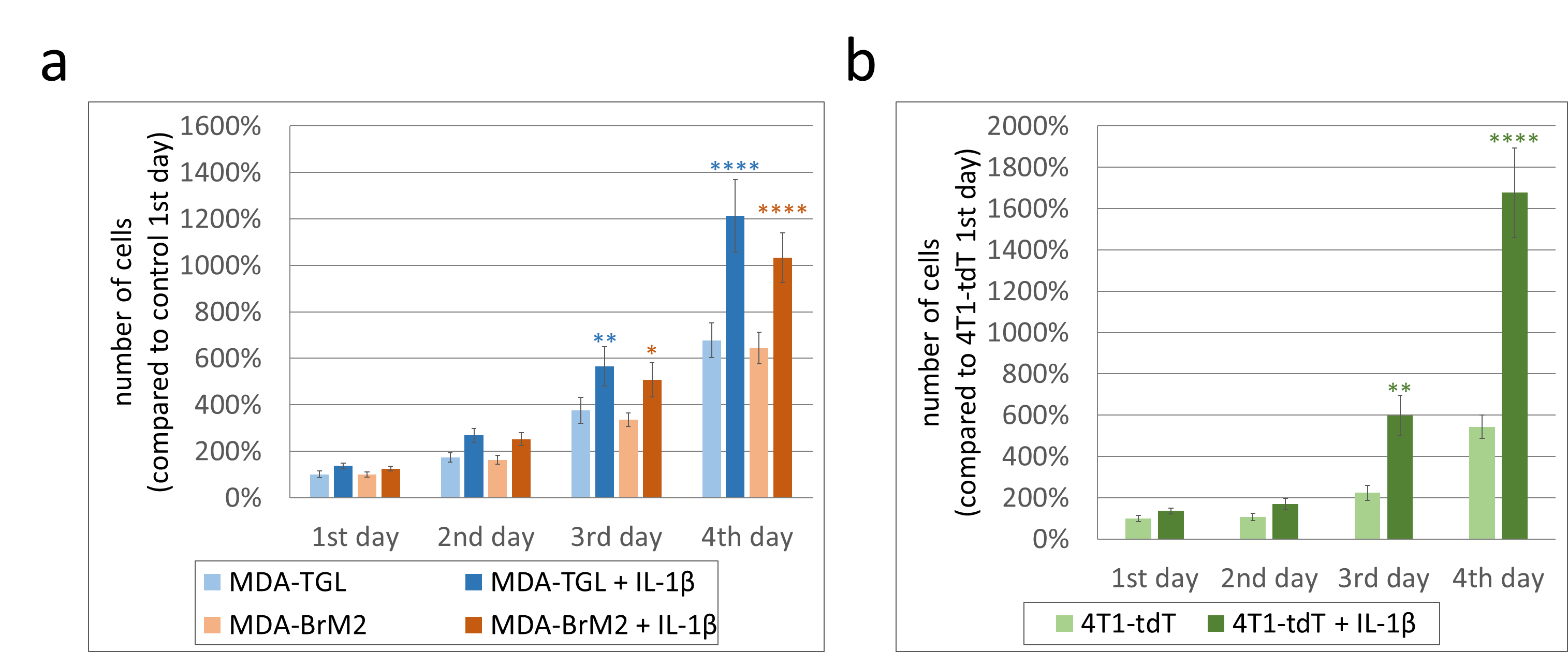


**Fig. S3. Proliferation of breast cancer cells in response to IL-1β. a**: Proliferation of human breast cancer cells in response to 10 ng/ml IL-1β. Graphs represent the average ± SEM (N = 3 independent experiments, each performed in duplicate, 5 different fields of view photographed from each well). * *P*≤0.05, ** *P*≤0.01, **** *P*≤0.0001 compared to control (MDA-TGL or MDA-BrM2, respectively) (ANOVA and Fisher's LSD *post hoc* test). **b**: Proliferation of mouse breast cancer cells in response to 10 ng/ml IL-1β. Graphs represent the average ± SEM (N = 3 independent experiments, each performed in duplicate, 5 different fields of view photographed from each well). ** *P*≤0.01, **** *P*≤0.0001 compared to control (4T1-tdT) (ANOVA and Fisher's LSD *post hoc* test).


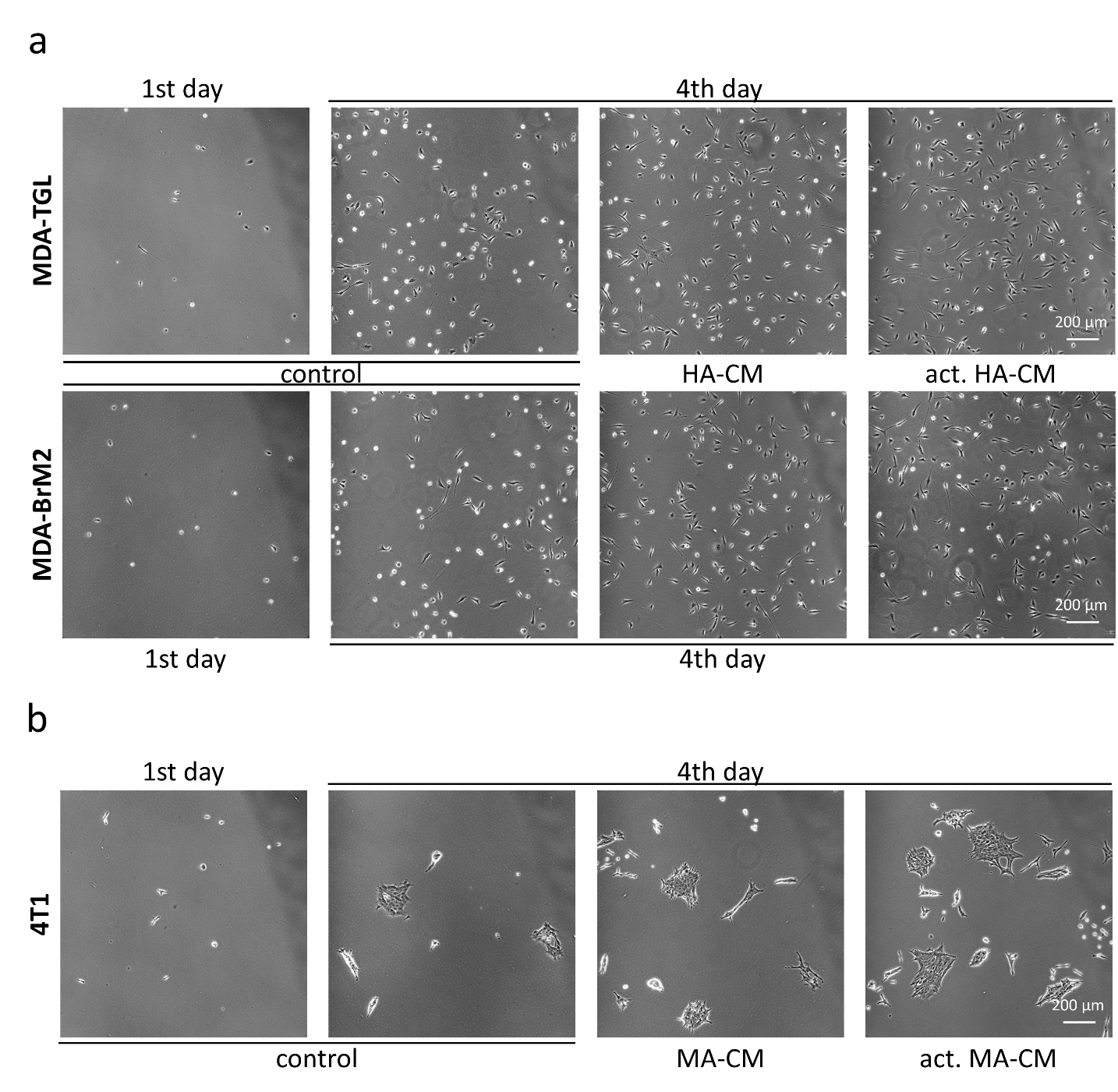


**Fig. S4. Proliferation of breast cancer cells in response to astrocyte-conditioned media. a**: Representative phase contrast images showing the proliferation of human breast cancer cells cultured in human astrocyte-conditioned media. **b**: Representative phase contrast images showing the proliferation of mouse breast cancer cells cultured in mouse astrocyte-conditioned media. Quantitative analyses are shown in **Fig. S5**.


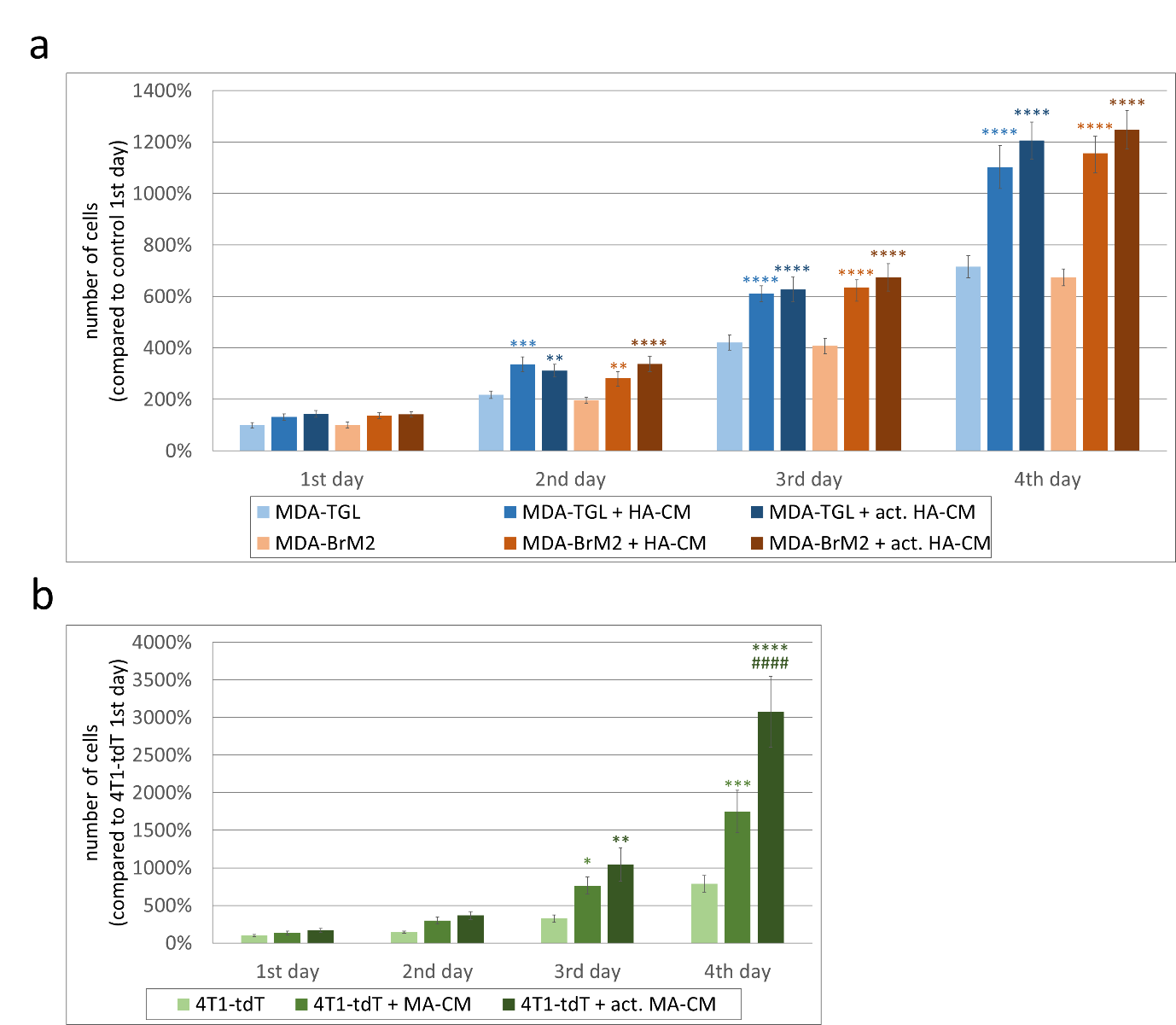


**Fig. S5. Proliferation of breast cancer cells in response to astrocyte-secreted factors (quantitative data). a**: Proliferation of human breast cancer cells cultured in human astrocyte-conditioned media. Graphs represent the average ± SEM (N = 3 independent experiments, each performed in duplicate, 5 different fields of view photographed from each well). ** *P*≤0.01, **** *P*≤0.0001 compared to the same day’s control (MDA-TGL or MDA-BrM2, respectively) (ANOVA and Fisher's LSD *post-hoc* test). **b**: Proliferation of mouse breast cancer cells cultured in mouse astrocyte-conditioned media. Graphs represent the average ± SEM (N = 3 independent experiments, each performed in duplicate, 5 different fields of view photographed from each well). * *P*≤0.05, ** *P*≤0.01, *** *P*≤0.001, **** *P*≤0.0001 compared to the same day’s control (4T1-tdT), #### *P*≤0.0001 compared to the same day’s “4T1-tdT + act. MA-CM” (ANOVA and Fisher's LSD *post hoc* test).


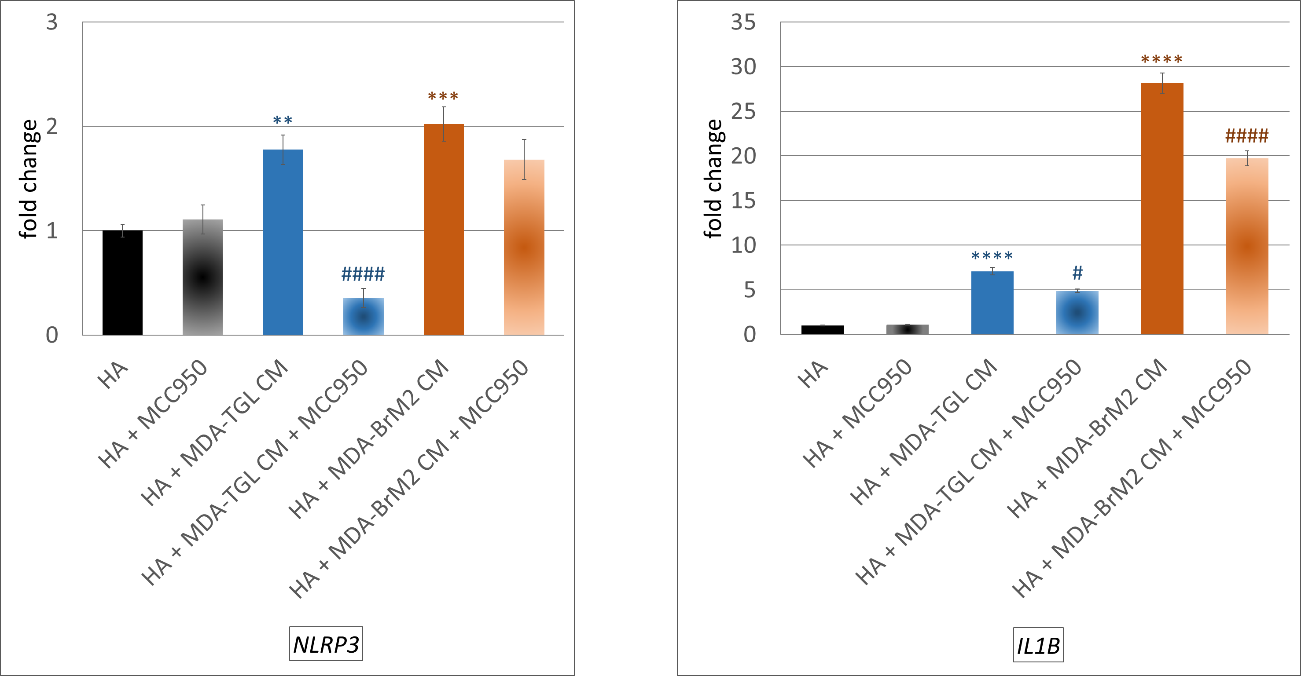


**Fig. S6. NLRP3 inhibition-induced changes in the expression of NLRP3 inflammasome-associated genes in astrocytes.** Expression of *NLRP3* and *IL1B* genes in human astrocytes cultured for 24 hours in human triple negative breast cancer cell-conditioned medium, in the presence or absence of 1 μM MCC950. Graphs represent the fold change (normalized to *GAPDH*), average ± SEM (N = 3 independent experiments, each performed in triplicate). ** *P*≤0.01, *** *P*≤0.001, **** *P*≤0.0001 compared to control (HA), # *P*≤0.05, #### *P*≤0.0001 compared to HA + MDA-TGL CM or HA + BrM2 CM, respectively (ANOVA and Fisher's LSD *post hoc* test).


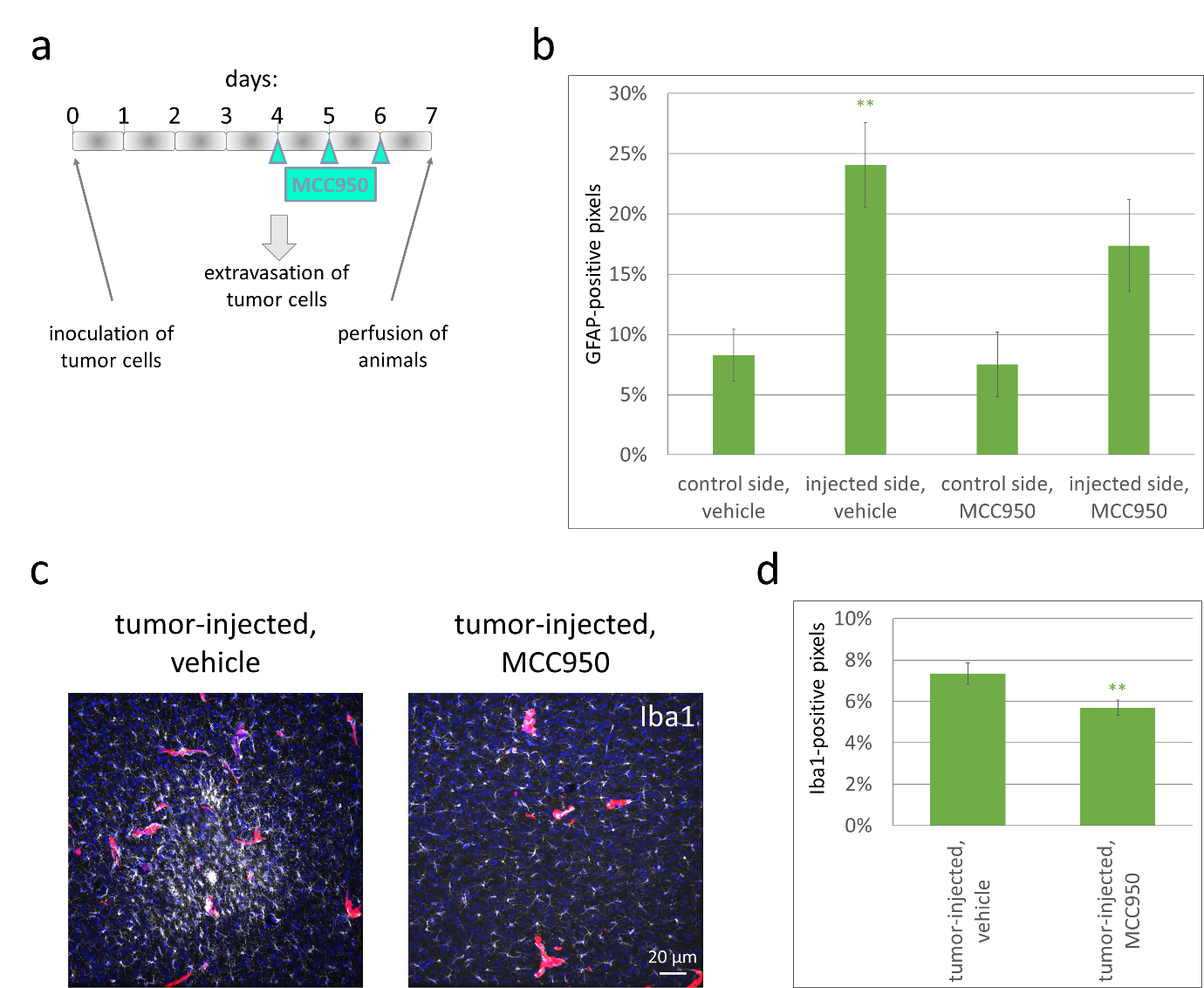


**Fig. S7. NLRP3 inhibition-induced reduction in gliosis associated with brain metastasis. a**: Schematic representation of the *in vivo* model (details presented in the **Materials and methods**). **b**: Percentage of GFAP-positive pixels compared to all pixels in brain sections of mice inoculated with 4T1-tdT triple negative breast cancer cells (tumor-injected) or modified Krebs-Ringer solution (control) and treated with vehicle (DMSO in PBS) or 10 mg/kg MCC950 7 days after the injection of the tumor cells. Graphs represent the average ± SEM (N = 3 independent experiments, n = 8 sections/animal). ** *P*≤0.01 compared to “control, vehicle” (ANOVA and Fisher's LSD *post hoc* test). **c**: Representative immunofluorescence micrographs (maximum intensity projections of *z*-stacks) showing a reduction in peritumoral Iba1 expression in response to systemic MCC950 treatment in mouse triple negative breast cancer brain metastases 7 days after inoculation of the tumor cells. **d**: Ratio of Iba1-positive pixels in MCC950-treated animals, compared to all pixels. Graphs represent the average ± SEM (N = 3 independent experiments, n = 6 sections/animal, 8 ROIs/6 sections). ** *P*≤0.01 (unpaired Student’s *t* test).
